# Quantification of AMPA receptor subunits and RNA editing-related proteins in the J20 mouse model of Alzheimer’s disease by capillary western blotting

**DOI:** 10.3389/fnmol.2023.1338065

**Published:** 2024-01-17

**Authors:** Luke T. Milham, Gary P. Morris, Lyndsey M. Konen, Peggy Rentsch, Nesli Avgan, Bryce Vissel

**Affiliations:** ^1^Centre for Neuroscience and Regenerative Medicine, St Vincent’s Centre for Applied Medical Research, St Vincent’s Hospital, Sydney, NSW, Australia; ^2^St Vincent’s Clinical School, Faculty of Medicine, University of New South Wales, Sydney, NSW, Australia; ^3^Tasmanian School of Medicine, College of Health and Medicine, University of Tasmania, Hobart, TAS, Australia

**Keywords:** RNA editing, Alzheimer’s disease, ADAR2, ADAR1, GluA1-4, capillary western blotting, AMPA receptor, J20 mouse model

## Abstract

**Introduction:**

Accurate modelling of molecular changes in Alzheimer’s disease (AD) dementia is crucial for understanding the mechanisms driving neuronal pathology and for developing treatments. Synaptic dysfunction has long been implicated as a mechanism underpinning memory dysfunction in AD and may result in part from changes in adenosine deaminase acting on RNA (ADAR) mediated RNA editing of the GluA2 subunit of AMPA receptors and changes in AMPA receptor function at the post synaptic cleft. However, few studies have investigated changes in proteins which influence RNA editing and notably, AD studies that focus on studying changes in protein expression, rather than changes in mRNA, often use traditional western blotting.

**Methods:**

Here, we demonstrate the value of automated capillary western blotting to investigate the protein expression of AMPA receptor subunits (GluA1-4), the ADAR RNA editing proteins (ADAR1-3), and proteins known to regulate RNA editing (PIN1, WWP2, FXR1P, and CREB1), in the J20 AD mouse model. We describe extensive optimisation and validation of the automated capillary western blotting method, demonstrating the use of total protein to normalise protein load, in addition to characterising the optimal protein/antibody concentrations to ensure accurate protein quantification. Following this, we assessed changes in proteins of interest in the hippocampus of 44-week-old J20 AD mice.

**Results:**

We observed an increase in the expression of ADAR1 p110 and GluA3 and a decrease in ADAR2 in the hippocampus of 44-week-old J20 mice. These changes signify a shift in the balance of proteins that play a critical role at the synapse. Regression analysis revealed unique J20-specific correlations between changes in AMPA receptor subunits, ADAR enzymes, and proteins that regulate ADAR stability in J20 mice, highlighting potential mechanisms mediating RNA-editing changes found in AD.

**Discussion:**

Our findings in J20 mice generally reflect changes seen in the human AD brain. This study underlines the importance of novel techniques, like automated capillary western blotting, to assess protein expression in AD. It also provides further evidence to support the hypothesis that a dysregulation in RNA editing-related proteins may play a role in the initiation and/or progression of AD.

## Introduction

Alzheimer’s disease (AD) dementia is a complex cognitive disorder associated with synaptic dysfunction, loss of dendritic spines and neuronal loss (Honer et al., 1992; Gómez-Isla et al., 1997; Coleman and Yao, 2003; Scheff et al., 2014). The causes of AD are currently unclear, with many genetic and non-genetic risk factors potentially contributing to disease pathogenesis (Nixon, 2017; Gong et al., 2018; Morris et al., 2018).

Among the changes known to occur in AD, altered glutamatergic neurotransmission, critical to synapse function, has long been implicated in AD pathogenesis (Greenamyre et al., 1988; Hynd et al., 2004; Bukke et al., 2020). Glutamate acts on several receptors in the brain, one of which is the ionotropic alpha-amino-3-hydroxy-5-methylisoxazole-4-propionic acid receptor (AMPAR). AMPARs are formed through heteromeric tetramers of four protein subunits, GluA1-GluA4 (Rossmann et al., 2011; Gan et al., 2016; Konen et al., 2020). The differing combinations of these subunits diversify AMPAR physiochemical properties (Hollmann et al., 1991; Verdoorn et al., 1991; Youn et al., 2008). For example, AMPARs lacking GluA2 are Ca^2+^-permeable and AMPARs containing GluA2/GluA3 homodimers can have different functional properties to GluA1/2 AMPARs including increased activation during cAMP signalling as well as trafficking to the synapse (Hume et al., 1991; Isaac et al., 2007; Rossmann et al., 2011; Italia et al., 2021).

The links between AD pathology and AMPAR subunit dysregulation are numerous but sometimes conflicting. Some studies have found reductions in GluA2 and GluA2/3 within the hippocampus of AD patients (Carter et al., 2004), whereas others have found no changes in the expression of all four subunits in the same region (Hyman et al., 1994). Alternatively, in the dentate gyrus and cerebral spinal fluid (CSF) of AD patients, studies have found an upregulation of GluA2 (Yeung et al., 2021) and GluA3 (Enache et al., 2020) respectively. In the 3xTg mouse model of AD, there are age-dependent decreases in the levels of all subunits, except for GluA2 (Cantanelli et al., 2014). The causes and consequences of these changes are poorly understood. However, there is evidence some of these alterations may be compensatory. For example, GluA3 has been found to negatively regulate Aβ-dependent toxicity and rescue memory impairments in the APP/PS1 mouse model of AD (Reinders et al., 2016).

AMPAR function is also tightly regulated by a process known as RNA editing. RNA can undergo over 100 different biochemical modifications, collectively termed the epitranscriptome, which diversifies the transcriptome (Saletore et al., 2012; Helm and Motorin, 2017). One of these alterations, RNA editing, is a process whereby mRNA transcripts can be modified by the insertion, deletion, or substitution of nucleotides, which can alter the amino acid code and therefore the resulting function of proteins (Maas et al., 2006; Christofi and Zaravinos, 2019; Gassner et al., 2021). A particularly well-known example of this process is RNA editing at the Q/R site of GluA2 in which a CAG codon is converted to a CGG at position 608 in the mRNA. In healthy hippocampal neurons, this site is edited at >99% efficiency (Yamashita et al., 2012; Yamashita and Kwak, 2014; Pachernegg et al., 2015) and receptors containing edited GluA2 are Ca^2+^-impermeable due to the presence of a positively charged arginine (R) within the pore-lining region of the AMPAR. Conversely, receptors containing unedited GluA2 are Ca^2+^-permeable due to the presence of an uncharged glutamine (Q) (Sommer et al., 1991; Takuma et al., 1999; Wright and Vissel, 2012; Chaytow et al., 2021). Several studies have found significant decreases in the efficiency of GluA2 Q/R site editing in human AD brains (Akbarian et al., 1995; Gaisler-Salomon et al., 2014; Khermesh et al., 2016; Annese et al., 2018) which may cause a pathological change in the function of AMPARs via excitotoxicity (Van Damme et al., 2002; Vieira et al., 2010; Sebe et al., 2017). Evidence that a decrease in the efficiency of GluA2 Q/R site editing is pathological comes from several mouse studies showing that a reduction in GluA2 Q/R site editing leads to seizures, neurodegeneration, and impairments in memory (Brusa et al., 1995; Feldmeyer et al., 1999; Konen et al., 2020). Notably, we recently found that several pathological phenotypes of J20 mice could be rescued by crossing them with a line expressing a genetic modification that forcibly induces editing at the GluA2 Q/R site, highlighting the potential importance of editing at this site to the development of AD pathology (Wright et al., 2023).

Beyond the GluA2 Q/R site, large-scale transcriptomic studies have found alterations to the efficiency of RNA editing processes in AD for many neuronal transcripts containing conserved editing sites (Khermesh et al., 2016; Annese et al., 2018; Gardner et al., 2019; Ma et al., 2021). It is not yet clear why the efficiency of RNA editing might be affected in AD, but one likely upstream cause may be dysregulation of the adenosine deaminases acting on RNA (ADAR) enzymes responsible for the most common form of editing, adenosine to inosine (A-to-I). There are three ADAR proteins expressed in mammals: ADAR1, ADAR2, and ADAR3 (Maas et al., 2006; Savva et al., 2012). ADAR1 has two functional isomers, both with strong links to inflammatory and cancer pathologies (Mannion et al., 2014; Gannon et al., 2018; Xu and Öhman, 2019; Ramírez-Moya et al., 2020; Guo et al., 2021). The longer ADAR p150 isoform is regulated by (and suppresses) several inflammatory pathways related to interferon signalling (George et al., 2016; Li et al., 2021), while the shorter ADAR1 p110 isoform is constitutively expressed and has been implicated in mechanisms of cellular development/proliferation (Nemlich et al., 2013; Yang et al., 2015). ADAR2 is the primary mediator of GluA2 Q/R site editing (Liu and Samuel, 1999), has an essential role in synaptic plasticity (Vissel et al., 2001), and is fundamental in the early stages of brain development (Higuchi et al., 2000; Jacobs et al., 2009). Little is known about the functional role of ADAR3 other than that it is catalytically inactive, highly localised to the brain, and has been theorised to act as a competitive inhibitor of ADAR1 and ADAR2 (Mladenova et al., 2018; Kurup et al., 2022).

Several studies have investigated the expression of ADARs in AD (Akbarian et al., 1995; Gaisler-Salomon et al., 2014; Khermesh et al., 2016; Annese et al., 2018; Ma et al., 2021). ADAR2 has been shown to be downregulated in the caudate nucleus (Gaisler-Salomon et al., 2014) and prefrontal cortex (Ma et al., 2021) in AD but upregulated in the temporal lobe (Khermesh et al., 2016). Similarly, ADAR p110 is both up and downregulated in the frontal and temporal lobes, respectively, with concurrent upregulation of the ADAR1 p150 isoform in the hippocampus (Khermesh et al., 2016). Interestingly, ADAR3 has also been found to be upregulated in both the hippocampus (Annese et al., 2018) and prefrontal cortex (Ma et al., 2021) of AD patients. Furthermore, cytoplasmic mislocalisation of ADAR2 has been observed in brain tissue derived from both amyotrophic lateral sclerosis (ALS) and dementia patients (Moore et al., 2019). Collectively, these studies suggest ADAR enzymes become dysregulated in AD and that this may trigger changes in the efficiency of RNA editing.

The mechanistic causes of changes in ADAR1-3 expression, localisation and function in AD are unknown, although there are several reported molecular mechanisms by which ADAR-regulated RNA editing can be influenced. Increased phosphorylation of isomerase peptidyl-prolyl *cis-*trans isomerase NIMA-interacting 1 (PIN1) positively regulates the nuclear localisation of ADAR2 and the subsequent probability of mRNA binding (Marcucci et al., 2011). Alternatively, when localised outside the nucleus, ADAR2 is poly-ubiquitinated by WW Domain Containing E3 Ubiquitin Protein Ligase 2 (WWP2) leading to its subsequent degradation (Marcucci et al., 2011). Similarly to PIN1, fragile X-related protein 1 (FXR1P) has also been shown to alter the ability of ADARs to bind their target mRNA editing sites (Filippini et al., 2017; Tran et al., 2019). Finally, the transcription factor cAMP responsive element binding protein 1 (CREB1) can induce ADAR2 expression and, in turn, regulate ADAR2 target mRNAs (Peng et al., 2006). The involvement of these proteins in both ADAR-related RNA-editing processes and AD pathology has been demonstrated to various extents (Pláteník et al., 2014; Wang et al., 2020; Song et al., 2023; Zhou et al., 2023). Nevertheless, the expression of these proteins in an AD mouse model and the exact nature of the relationship between these proteins, particularly in the context of ADAR-mediated pathogenesis, remains unexplored.

There are relatively few reports detailing the protein expression of the GluA1-4 subunits and ADAR1-3 enzymes in mouse models of AD. Furthermore, there are, to the best of our knowledge, no studies investigating the expression of molecules that may influence ADAR function in mouse models of AD (e.g., PIN1, WWP2, FXR1P, CREB), alongside ADAR proteins. In this study, we therefore aimed to investigate the expression of these proteins in the J20 mouse model of AD. While the J20 model has a well-characterised progression of pathological processes exhibiting pathological and behavioural changes similar to those occurring in human AD (Wright et al., 2013; Ameen-Ali et al., 2019; Whitesell et al., 2019), the expression of AMPAR subunits and RNA editing-associated proteins have not been well characterised in this model.

There are a variety of techniques available to assess the expression of mRNA and proteins in tissue including autoradiography, immunoblotting, immunohistochemistry, *in situ* hybridisation, quantitative PCR and RNA sequencing. In this study, we provide the first report using capillary western blotting to quantify expression changes in AMPAR subunits and RNA editing proteins in a mouse model of AD. The relative novelty of this technology means there are few reports of systematic normalisation and optimisation of protocols relevant to its use (Castle et al., 2019; Sormunen et al., 2023). A second major aim of this study was therefore to provide a methodology to optimise and validate the capillary western system in order to detect changes in protein expression in a disease state.

## Materials and methods

### Mice

All animal work was performed with the approval of the Garvan Institute and St. Vincent’s Hospital Animal Ethics Committee, in accordance with the National Health and Medical Research Council animal experimentation guidelines and the Australian Code of Practice for the Care and Use of Animals for Scientific Purposes (2013). Hemizygous transgenic (J20) and non-transgenic littermates (wildtype; WT) were used from the B6.Cg-Tg (PDGFB-APPSwInd)20Lms/2J (J20) line. These J20 mice (sometimes referred to as hAPP-J20) (Wright et al., 2013) overexpress mutant APP (Amyloid precursor protein) containing both the Swedish and Indiana mutations, under the control of the PDGF-β chain promoter. Mice were maintained on a C57BL/6J background. Mice were group housed on a 12 h light/dark cycle with *ad libitum* access to food and water. For all experiments, mice were acclimatised for at least 1 week in holding rooms, before being used in experimental procedures. A total of 40 male mice were used throughout the study.

### Barnes maze setup

The apparatus comprised a circular, white platform measuring 920 mm in diameter, positioned at a height of 1 m above the ground. The perimeter of the platform contained 20 identical holes that were equally spaced. A black escape box, measuring 175 mm (D) × 75 mm (W) × 80 mm (H), was hidden beneath one of the holes, while the other holes were blocked. Mice were subjected to negative reinforcements (bright lights and loud noises) and were expected to use the visual cues located in the room to find the hidden escape box. The activity of the animals was recorded using the ANYmaze Video Tracking System 6.33 (Stoelting Co.) with a DMK 22AUC03 camera placed directly above the maze.

### Barnes maze testing

44-week-old mice underwent three phases during the Barnes Maze protocol: habituation, acquisition, and the probe (test) trial. During the 2 min habituation phase, mice were placed in the centre of the platform and covered with a glass beaker and moved slowly toward the escape box to learn its location. The acquisition phase consisted of 3 training trials per day with a 35–45 min interval between each trial, for 5 days. During these trials, the mice were placed in a cylindrical chamber measuring 80 mm in diameter and 12.5 mm in height, positioned at the centre of the maze and facing a random direction. The chamber was then lifted, and the mice were given 2 min to locate the hidden escape box. If the animals were unable to locate the escape box within the given time, they were manually guided to it. The location of the escape box was randomly assigned to each animal before the experiment, but it remained constant for each individual mouse throughout the trial. The probe trial was conducted 24 h after the last acquisition trial, during which the hidden escape box was removed, and its hole was undifferentiated from the other holes on the platform. Mice were given 60 s on the platform, during which they were expected to move to the location where the escape box was previously located. The results measured for each mouse included primary latency, primary distance, and primary errors.

### Hippocampal dissection and tissue preparation

A separate cohort of 44-week-old mice were anesthetised with isoflurane and cervically dislocated. Whole brains were extracted and placed on a sterile plate cooled with ice. The hippocampus was rapidly dissected and removed from both the left and right cerebral hemispheres. The extracted hippocampal tissue was placed within sterile Eppendorf tubes and snap-frozen in dry ice. Samples were then stored at −80°C before being processed for protein extraction. For all experiments performed within this study, only the right hippocampus was used.

### Protein extraction

Hippocampal tissue was suspended in 200 μl of RIPA buffer (Sigma-Aldrich, Cat# MFCD02100484) containing Protease Inhibitor Cocktail (Sigma-Aldrich, Cat#S8830) and Phos-stop dephosphorylation inhibitors (Sigma-Aldrich, Cat#4906845001). Tissue was homogenised by sonication and the resulting cell suspension was centrifuged at 14,000 rpm for 15 min at 4°C and supernatant was collected. If being used immediately, protein extracts were placed on ice. Otherwise, extracts were aliquoted and stored at −80°C until required. Each aliquot was freeze-thawed no more than 3 times to ensure their integrity.

### Bradford assay

The concentrations of all protein samples were quantified in technical triplicates utilising a Bradford assay. A standard curve was generated with Pre-Diluted Bovine Serum Albumin (ThermoFisher, Cat# 23208) by performing standards in triplicate across a dilution range of 0–2000 μg/ml. The concentrations of unknown samples were interpolated from this curve according to the manufacturer’s instructions. Absorbance was measured using a FLUOstar Omega Microplate reader (BMG-Labtech). Concentrations were determined by interpolating the standard curve.

### WES automated capillary western blotting

Optimal antibody concentrations and linear dynamic ranges for each target protein were determined prior to conducting expression analyses. To quantify target proteins in J20 mice compared with WT littermates, samples were run on a standard 24-well WES operating plate, as per the manufacturer’s instructions. Reagents were obtained from 12 to 230 kDa separation modules (ProteinSimple Cat#SM-W004) and total protein detection modules (ProteinSimple Cat#DM-TP01). For experiments investigating the expression of target proteins, samples were pipetted in two separate wells within the plate. An internal control sample (derived from a WT littermate) was run in technical duplicates for both the primary antibody targets and total protein concentration so that data could be standardised to this internal calibrator across multiple plates. Several wells were used to run controls on each plate including controls for background biotinylation in sample diluent in the absence of sample, background biotinylation in the sample in the absence of the biotinylation label and background antibody signal within the sample diluent in the absence of sample.

### Analysis of capillary western data

Data was analysed using the WES instrument software (ProteinSimple, Compass for SW 4.1 Windows 7/8/10 64 bit). Peak analysis settings were performed as follows: Range: (1–250); Baseline: Threshold (0.1), Window (400), Stiffness (0.1); Peak Find: Threshold (10), Width (9), Area Calculation (Dropped lines). Baseline adjustments were made to fit relative background chemiluminescence signals with all samples measured at identical conditions. Dropped line analysis was preferred over a Gaussian fit model to adjust for interfering additional peaks and for better control of the relative peak signal. Supplementary Table 1 describes antibodies measured in WES analysis and peaks recorded for antibodies used in the procedure.

### Statistics

Statistical analyses were performed using GraphPad Prism 8.2.1 (441). Outliers were identified and excluded using Grubbs’ test for outliers (α = 0.05). The D’Agostino-Pearson test was employed to assess normality and lognormality, in order to determine whether the data were parametric or non-parametric. For parametric groups, an *F*-test of variance was conducted to ascertain whether standard deviations were equal between individual data sets. In cases where standard deviations were equal, a student’s *t*-test was utilised to determine significance between the two groups. Conversely, for groups with unequal standard deviations, Welch’s *t-*test was applied. For non-parametric groups, the Mann-Whitney test was used to determine the significance between the two groups. Statistical significance was defined as *p* < 0.05 and denoted as *p < 0.05, **p < 0.01, ***p < 0.001, ****p < 0.0001. Data are presented as mean ± SD unless otherwise specified. For the quantification of whole hippocampal GluA1-4 protein expression, as well as ADAR1-3, FXR1P, WWP2, PIN1, and CREB1 protein expression, a sample size of *n* = 12 per group was utilised. Due to the novelty of these experiments, the expected means, standard deviations, and effect sizes were unknown. The maximum sample size available for these initial investigations was *n* = 12. The data supporting the findings of this study are provided in a Supplementary document.

## Results

### Optimisation of loading controls and linear range of the capillary western blotting system

Prior to analysing protein expression in the hippocampus of J20 mice, we first conducted a series of experiments to optimise our automated capillary western protocols. Identical to traditional western blotting, protein quantification can be determined by normalising target protein expression levels to loading controls. In traditional western blotting, these loading controls have predominantly been “housekeeping” proteins (e.g., β-Tubulin, GAPDH, and β-Actin) (Li and Shen, 2013). However, although housekeeping proteins are thought to be expressed consistently within tissue under disease conditions, research now suggests this may not always be the case (Ferguson et al., 2005; Lin and Redies, 2012). In the context of neurological diseases, several studies have demonstrated that commonly used housekeeping proteins do in fact fluctuate in disease conditions (Li and Shen, 2013; Vigelsø et al., 2015). To overcome these issues, it has become common to normalise target protein expression to the total protein expression of a given sample (Aldridge et al., 2008; Kirshner and Gibbs, 2018).

**FIGURE 1:**
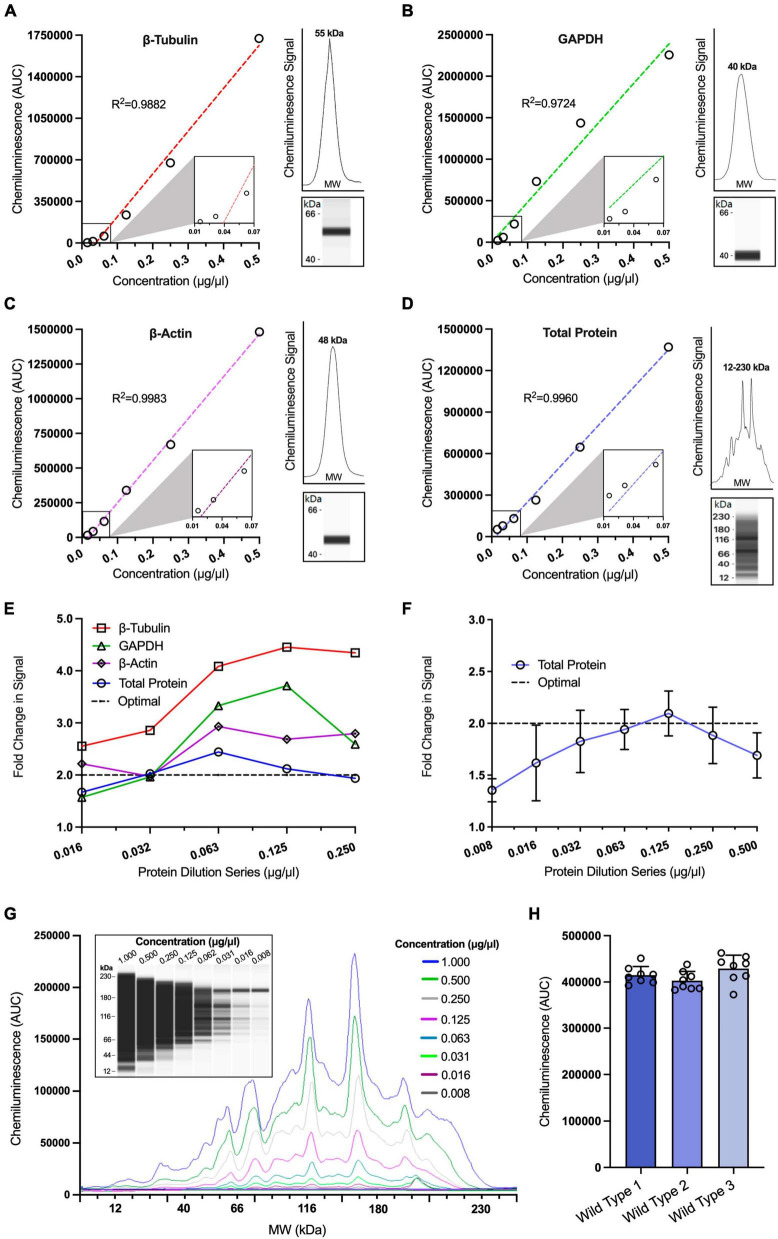
Optimisation of loading controls in capillary western blotting. Simple linear regression analysis of chemiluminescence signals obtained from a twofold protein dilution series for β-Tubulin [**(A)**; *r*^2^ = 0.9882], GAPDH [**(B)**, *r*^2^ = 0.9724], β-Actin [**(C)**; *r*^2^ = 0.9983] and total protein [**(D)**; *r*^2^ = 0.9960] (*n* = 1 for all dilutions). Insets situated to the right of graphs **(A–D)** display the electropherogram peak (above) with a computer-generated blot (below) for the target protein analysed. **(E)** Comparison of relative fold change in chemiluminescence signals for a twofold protein dilution series assessing three housekeeping proteins, GAPDH, β-Tubulin and β-Actin against total protein. Dotted line represents the theoretical optimal result when the chemiluminescence is halved for visual comparison. **(F)** Expanded range of total protein dilution series and analysis of fold change in chemiluminescence signals across multiple replicates (*n* = 6–7). Dotted line represents theoretical optimal result for visual comparison. **(G)** Representative capillary western data of twofold total protein dilution series using a chemiluminescence electropherogram and a corresponding computer-generated blot. **(H)** Comparison of mean total protein chemiluminescence signal analysed across three different wild type (WT) hippocampal samples, each of which was run in 8 technical replicates at a concentration of 0.125 μg/ml on the same 24-well plate (*n* = 8), one-way ANOVA *p* > 0.05, D’Agostino and Pearson test *p* > 0.05. All figures are mean ± SD.

To determine the appropriate loading controls for our study, we used linear regression to assess the relationship between signal intensity and concentration for a range of traditional housekeeping proteins and compared these to the total protein expression of each sample. For all housekeeping proteins, we used saturating concentrations of antibodies for detection (0.5 μg/ml). We observed a strong linear relationship between signal intensity and protein concentration for all housekeeping proteins and the total protein assay (Figures 1A–D: β-Tubulin *r*^2^ = 0.9882, GAPDH *r*^2^ = 0.9724, β-Actin *r*^2^ = 0.9983, total protein *r*^2^ = 0.9960). Another way to illustrate this is to quantify the fold change in signal after each twofold dilution, which should theoretically halve each time. This approach allowed us to visually compare traditional housekeeping proteins to the total protein assay (Figure 1E). Using this approach, it was noticeable that the signals from the housekeeping proteins were not following a twofold decreasing trend in signal intensity at each twofold dilution. In fact, we observed that both GAPDH and β-Actin signals did not fall within the twofold range until the sample protein concentration decreased to 0.032 μg/ml. Furthermore, β-Tubulin exhibited signal values that were outside the twofold range at all dilutions, suggesting a non-linear relationship within these concentration ranges. This poses a challenge when comparing the signal of these proteins to weakly expressed proteins that may exhibit a linear decrease in signal at higher protein concentrations. In contrast, our findings revealed that the total protein assay demonstrated a linear decrease of twofold across a wider range of concentrations (0.032–0.250 μg/ml), suggesting the possibility of normalising data to total protein even for weakly expressed proteins.

To confirm that the total protein assay provided a strong linear relationship between signal and protein concentration, we repeated the total protein assay over a larger number of replicates (*n* = 6), finding that the assay was consistently linear within the ranges 0.032–0.250 μg/ml (Figures 1F, G). These findings illustrate that the total protein assay is suitable for normalisation over a wide range of protein concentrations. These concentrations were subsequently used as the starting point when designing assays to test the linear range of target proteins so that both our target proteins and the total protein assay were assessed within their linear range.

Finally, we determined the reproducibility of the total protein assay across multiple technical and biological replicates. One downside of non-fluorescent capillary western blotting is that the total protein assay cannot be multiplexed with other antibodies, as the chemiluminescence signal from the total protein assay would obscure the signal from other antibodies. Therefore, to compare the expression levels of our proteins of interest to the total protein expression within a sample, it was necessary to determine the signal variance when a single sample is pipetted multiple times.

We conducted a total protein assay on hippocampal tissue from 3 WT mice, each of which was analysed across 8 technical replicates at 0.064 μg/ml. There was no significant difference found in the signal between the 3 WT samples (Figure 1H). Furthermore, the coefficient of variance was <10% for each biological replicate (4.50, 4.98, and 6.76%), a result similar to those found in another study investigating the efficiency of capillary western blotting (Rustandi et al., 2012) and less than the reported variability found in traditional western blotting of >35% (Koller and Wätzig, 2005). This provided confidence we could accurately normalise our target protein signals to the total protein signal.

### Optimising linear range and antibody concentrations for proteins of interest

**FIGURE 2:**
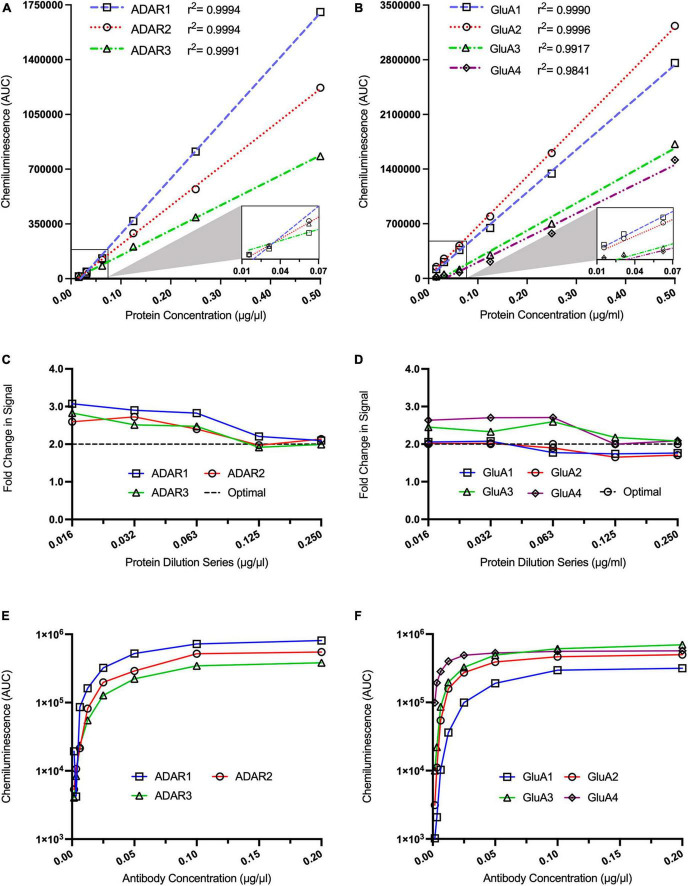
Optimisation of ADAR and GluA subunit antibody concentrations for automated capillary western blotting. Simple linear regression analysis of ADAR1 [**(A)**, *r*^2^ = 0.9994], ADAR2 [**(A)**, *r*^2^ = 0.9994], and ADAR3 [**(A)**, *r*^2^ = 0.9991] as well as GluA1 [**(B)**, *r*^2^ = 0.9990], GluA2 [**(B)**, *r*^2^ = 0.9996], GluA3 [**(B)**, *r*^2^ = 0.9917] GluA4 [**(B)**, *r*^2^ = 9841] chemiluminescence signals obtained from a twofold protein dilution series (*n* = 1 for all dilutions). **(C)** Analysis of fold changes in ADAR1, ADAR2, and ADAR3 chemiluminescence signals across a twofold dilution series (*n* = 1). **(D)** Analysis of fold changes in GluA1, GluA2, GluA3, and GluA4 chemiluminescence signals across a twofold dilution series (*n* = 1). Dotted lines in **(C,D)** represent the theoretical optimal result when the chemiluminescence is halved for visual comparison. Chemiluminescence signals from ADAR1, ADAR2, and ADAR3 antibodies **(E)** as well as from GluA1, GluA2, GluA3, GluA4 antibodies **(F)** over a twofold dilution series (*n* = 1 for all dilutions).

We next assessed the linear range of our target proteins to determine the optimal protein concentration for each target. To do this, we conducted a six-point, twofold dilution of protein derived from a calibrator sample. For all target proteins, the antibody concentration was run at a saturating dilution of 0.5 μg/ml. Similar to the housekeeping proteins, a strong linear relationship was displayed for the ADAR proteins (Figure 2A: ADAR1 *r*^2^ = 0.9994, ADAR2 *r*^2^ = 0.9994, ADAR3 *r*^2^ = 0.9991) and GluA subunits (Figure 2B: GluA1 *r*^2^ = 0.9990, GluA2 *r*^2^ = 0.9996, GluA3 *r*^2^ = 0.9917, GluA4 *r*^2^ = 0.9841) between signal intensity and protein concentration. Specifically, ADAR1-3 were linear between 0.125 and 0.250 μg/ml (Figure 2C). This was also the case for the AMPA subunits GluA3 and GluA4 (Figure 2D). GluA3 and GluA4 are relatively lowly expressed in comparison to GluA1 and GluA2, likely explaining why GluA1 and GluA2 were still linear at the lowest tested concentrations (between 0.016 and 0.063 μg/ml; Figure 2D). In subsequent quantification experiments, we adopted these ranges when assessing each protein to ensure we were working within the linear range of each protein.

Following this, we assessed the saturation point for each antibody to ensure antibody binding was not a rate-limiting step in the assay. For each target protein, the protein concentration was within the linear range of the target, based on our previous experiments (i.e., ADAR1-3 and GluA3-4: 0.250 μg/ml; GluA1-2: 0.063 μg/ml). ADAR1-3 antibodies all reached saturation at >0.1 μg/ml (Figure 2E), whereas the GluA1-4 antibodies reached a plateau at >0.05 μg/ml (Figure 2F). Antibody concentrations above these saturation points were subsequently used for each assay. Optimisation of protein concentrations and antibody saturation points were similarly conducted for all other target proteins PIN1, WWP2, FXR1P, and CREB (Supplementary Figures 1, 2).

Notably, the ADAR1 antibody used in this study binds both p110 and p150 isoforms. Due to the significantly higher expression of p110 in the brain and the larger peak observed in the electropherogram traces obscuring p150 at lower concentrations, optimisations were performed solely on the p110 peak. This may impact the accuracy of p150 quantification and should be noted in regard to the conclusion obtained from its analysis. Representative examples of original, uncropped capillary western blot traces for all proteins analysed in this paper in WT and J20 tissue are included in Supplementary Figures 3–8.

### Expression of GluA3 and ADAR1 p110 increased while ADAR2 decreased in 44-week-old J20 mice

**FIGURE 3:**
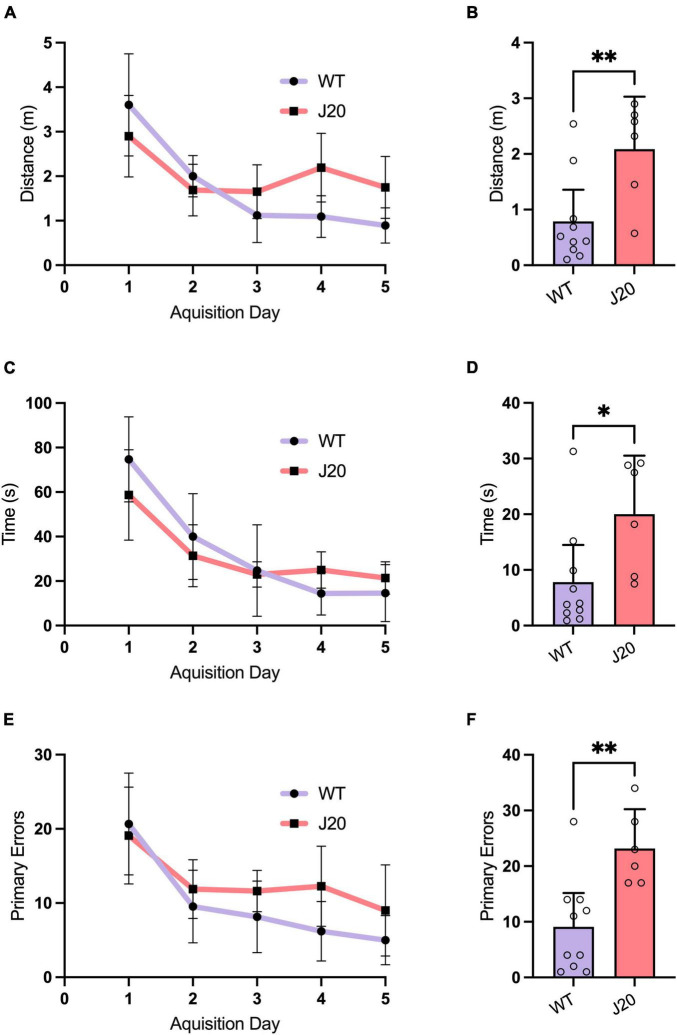
Barnes maze behaviour in 44-week-old J20 and WT mice. Acquisition learning curves showed that J20 mice exhibit normal spatial learning in the Barnes maze when compared to WT mice as they displayed similar performances in primary latency [**(A)**, 2-way repeated measures ANOVA, *p* = 0.1308], primary distance [**(C)**, 2-way repeated measures ANOVA, *p* = 0.1004] and primary errors [**(E)**, 2-way repeated measures ANOVA, *p* = 0.0955] in locating the escape box over the 5 days of acquisition training. During the Probe Trial, J20 mice displayed significant differences in primary latency [**(B)**, unpaired *t*-test *p* = 0.0276], primary distance [**(D)**, unpaired *t*-test *p* = 0.0092] and primary errors [**(F)**, unpaired *t*-test *p* = 0.0040] in finding the escape box location. For all graphs J20 *n* = 6 and WT *n* = 10. *p < 0.05, **p < 0.01. All figures are mean ± SD.

The J20 mouse is a widespread model of AD with well-characterised behavioural and histopathological alterations (Palop et al., 2003; Esposito et al., 2006; Galvan et al., 2006; Cheng et al., 2007; Harris et al., 2010; Cissé et al., 2011; Verret et al., 2012). In our own previous studies using J20 mice at similar, or younger ages than those used in this study, we have identified impaired devaluation performance in goal-directed action tests, spatial reference/working memory deficits in the radial arm maze, short-term memory deficits in a Y Maze and hyperactivity in an open field test. Despite this, no changes in anxiety on an elevated plus test, fear memory in a context fear conditioning paradigm, or novel object recognition in an object recognition task have been observed (Wright et al., 2013, 2023; Dhungana et al., 2023). To both expand upon the characterisation of the J20 mouse model and confirm our cohort had not experienced genetic drift, which has been shown to occur in AD mouse models (Cho et al., 2022), a separate cohort of J20 and WT mice underwent behavioural testing in a Barnes maze task, to identify if memory deficits were still present in our colony of J20 mice. As expected, both WT and J20 mice were able to learn the location of the escape box within the 5-day acquisition phase (Figures 3A, C, E). During the probe trial, J20 mice took significantly more time (Figure 3B: 7.800 ± 9.367 vs. 20.00 ± 10.03, *p* = 0.0276), travelled a significantly longer distance (Figure 3D: 0.7872 ± 0.7974 vs. 2.087 ± 0.8970, *p* = 0.0092), and made significantly more errors (Figure 3F: 9.100 ± 8.504 vs. 23.17 ± 6.735, *p* = 0.0040) to find the escape box location. These results confirmed that spatial memory deficits were still present in our J20 mice indicating a preserved AD phenotype.

**FIGURE 4:**
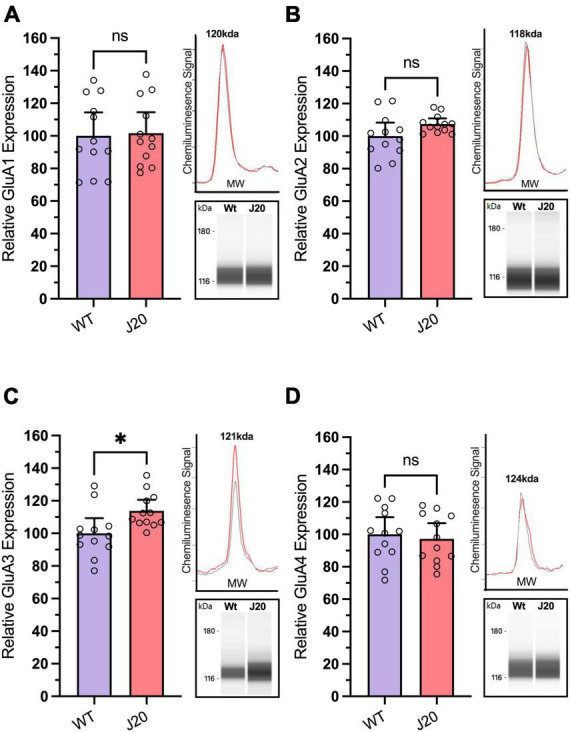
Analysis of GluA subunit protein expression in hippocampal tissue from J20 and WT mice. Relative expression of AMPA receptor subunits GluA1 [**(A)**; unpaired *t*-test *p* = 0.8514], GluA2 [**(B)**; Welch’s *t*-test *p* = 0.0899], and GluA4 [**(D)**; unpaired *t*-test *p* = 0.6776] remained unchanged in the hippocampus of 44-week-old J20 mice when compared to age-matched WT littermates. Although the expression of the GluA3 subunit [**(C)**; unpaired *t*-test *p* = 0.0214] is significantly upregulated. Insets situated right of all graphs display the electropherogram peak (top) with a computer-generated blot (bottom) for the related target protein analysed. *p < 0.05, ns = not statistically significance. All figures are mean ± SD.

To measure the expression of both AMPAR subunits and RNA-editing-related proteins in J20 mice, we extracted protein from the hippocampus of twelve 44-week-old J20 and 12 age-matched WT littermates. We observed no significant difference in the expression of GluA1 (Figure 4A: 100.0 ± 6.556% vs. 101.7 ± 5.827%, *p* = 0.8154), GluA2 (Figure 4B: 100.0 ± 3.773% vs. 95.36 ± 1.597%, *p* = 0.0899), or GluA4 (Figure 4D: 100.0 ± 4.806% vs. 95.36 ± 4.373%, *p* = 0.6776) in WT vs. J20 mice. However, there was a significant increase in the expression of the GluA3 subunit (Figure 4C: 100.0 ± 4.245% vs. 95.36 ± 3.061%, *p* = 0.0150) within the J20 cohort when compared to the WTs.

ADAR1 has two isoforms, ADAR1 p110 and ADAR1 p150, which have predicted molecular weights of ∼110 and ∼150 kDa, respectively. Two peaks corresponding to these molecular weights were observed following capillary western blotting with the ADAR1 antibody, allowing us to quantify the isoforms separately. Due to the relatively low expression of p150 compared to p110, it was necessary to verify the antibody was accurately detecting p150. Treatment of SH-SY5Y cells with type I and II interferons led to a moderate increase of p110 (≈20–50%) and a significant upregulation of p150 (≈300–500%) which was not mirrored following treatment with IL1-B, a cytokine not significantly linked to ADAR1 p150 (Supplementary Figure 9). Considering the p150 isoform is highly interferon-inducible (George et al., 2016; Li et al., 2021) this provided confidence that the p150 peak observed was accurate and could be used to quantify changes in mice.

**FIGURE 5:**
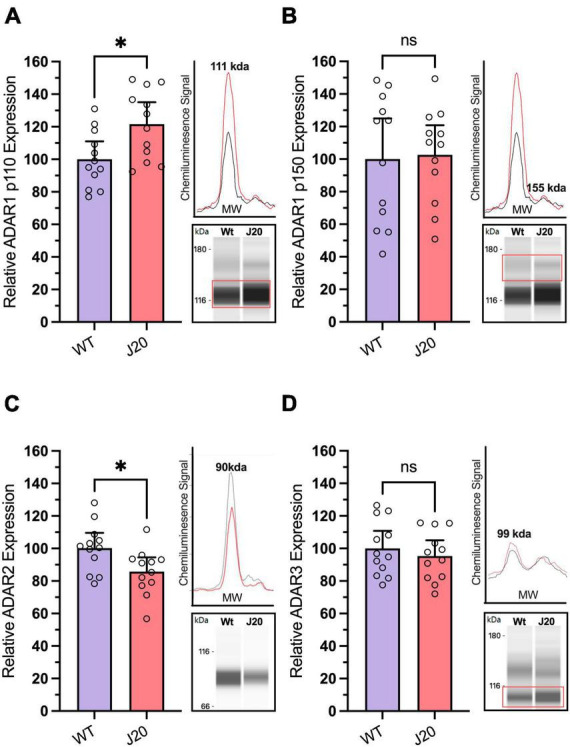
Analysis of ADAR protein expression in hippocampal tissue from J20 and WT mice. Relative expression of the ADAR1 p110 isoform [**(A)**; unpaired *t*-test, *p* = 0.0121] was significantly increased in the hippocampus of J20 mice compared to age-matched WT littermates, while no differences were observed in the ADAR1 p150 isoform [**(B)**; unpaired *t*-test, *p* = 0.8541] or in ADAR3 expression [**(D)**; unpaired *t*-test *p* = 0.4871]. Notably, ADAR2 protein expression was significantly reduced in J20 mice compared to controls [**(C)**; unpaired *t*-test *p* = 0.0214]. Insets situated to the right of all graphs display the electropherogram peak (top) with a computer-generated blot (bottom) for the target protein analysed. *N* = 12 for all groups. The target protein is highlighted within the red boxes. *p < 0.05, ns = not statistically significance. All figures are mean ± SD.

When performing our mouse experiments we found the expression of ADAR1 p110 was significantly increased by 21.8% in J20 mice compared to WTs (Figure 5A: 100.0 ± 4.9% vs. 121.8 ± 6.2%, *p* = 0.0121), but the expression of ADAR1 p150 was unchanged (Figure 5B: 100.0 ± 11.4% vs. 102.6 ± 8.3%, *p* = 0.8541). Notably, the expression of ADAR2 was found to be significantly decreased in J20 mice compared to WT controls (Figure 5C: 100.0 ± 4.669% vs. 85.08 ± 4.335%, *p* = 0.0296). Finally, the expression of ADAR3 was not significantly altered in J20 mice compared to WT mice (Figure 5D: means 100.0 ± 4.901% vs. 95.36 ± 4.366%, *p* = 0.4871). Collectively, this data suggests there are changes in the expression of key enzymes involved in A-to-I RNA-editing and AMPA receptor subunits in the hippocampus of J20 mice.

### J20-specific correlations found in the analysis of AMPAR subunits and RNA-editing linked proteins

**FIGURE 6:**
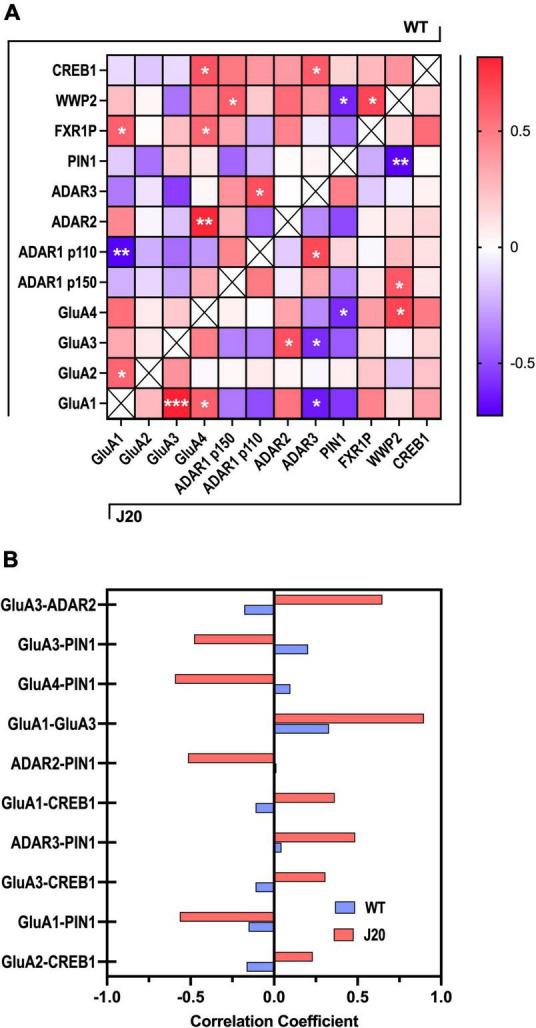
Correlation matrix of GluA subunits, ADAR, and RNA editing-associated proteins in J20 and WT hippocampal tissue. **(A)** A heatmap of the Pearson correlation coefficients between all target proteins analysed from J20 mice and age-matched WT littermates (*n* = 12 for each protein target). WT and J20 protein-protein associations are denoted in the top-left and bottom right half of the heat map, respectively. Positive correlations are indicated with increasing red colouration and negative correlations are indicated with increasing blue colouration. **(B)** Pearson correlation coefficients of the top 10 most differentially expressed protein-protein associations across WT and J20 mice. *p < 0.05, **p < 0.01, ***p < 0.001. All figures are mean ± SD.

Despite finding no significant changes in the overall expression of proteins PIN1, WWP2, FXR1, or CREB in the hippocampus of J20 mice (Supplementary Figure 10), a benefit of capillary western blotting is that little sample is used for each assay, allowing protein homogenates to be used for several assays. Considering we analysed the expression of multiple protein targets from the same hippocampal homogenates, it was feasible to conduct a *post hoc* regression analysis to assess the relationship between expression changes in these proteins across different samples (Supplementary Figure 11). We therefore performed a correlation matrix on the relative expression of the analysed proteins. Pearson correlation coefficients were determined for each protein pairing within both WT and J20 groups, with both genotypes exhibiting a combined total of 11 significantly correlated proteins (Figure 6A). Only four protein-protein correlations were shared between the two groups, indicating specific correlation patterns between WT and J20 mice. Of particular interest were the significant correlations within the J20s that were not observed within the WTs. This was distinctly seen in the AMPA subunits wherein GluA1-GluA3 (*r* = 0.82, *p* < 0.001), GluA1-GluA4 (*r* = 0.6, *p* = 0.039), GluA1-ADAR3 (*r* = −0.63, *p* = 0.025), GluA3-ADAR2 (*r* = 0.65, *p* = 0.023), GluA3-ADAR3 (*r* = −0.59 *p* = 0.042), GluA4-PIN1 (*r* = −0.62, *p* = 0.03), and GluA4-WWP2 (*r* = 0.70, *p* = 0.012) were all significantly correlated.

Furthermore, using the Pearson correlation coefficients obtained from the initial heat map a further analysis was performed to examine the protein-protein correlations that exhibited the largest differences between WT and J20 R-values (Figure 6B). Specific focus was placed on results wherein the J20 mice displayed larger variance in absolute correlation coefficient values relative to the WT controls. From this PIN1, CREB1, and the AMPAR subunits (specifically GluA3) showed the most consistent association with other RNA-editing related proteins within the J20s. Interestingly, the most significant variation between genotypes was found in the positive association of GluA3-ADAR2. This was unexpected as these proteins were found to be up and downregulated in the J20 mice, respectively. These observations suggest potential disparities in protein interaction networks between the two genotypes beyond the surface-level expression profile and the differences found in these correlations.

## Discussion

Previous studies have reported widespread dysregulation of RNA editing efficiency of numerous RNAs in human AD as well as changes in the expression of ADAR1-3 that are collectively responsible for RNA editing (Akbarian et al., 1995; Gaisler-Salomon et al., 2014; Khermesh et al., 2016; Annese et al., 2018; Ma et al., 2021) and apparent mislocalisation of ADAR2 to the cytoplasm (Moore et al., 2019). The mechanistic causes underlying these changes remain unknown.

In this study, we optimised the automated capillary western blotting method, showing an approach for normalising to total protein and illustrating how to characterise optimal protein/antibody concentrations for accurate quantification of proteins. In this study we specifically focused on the AMPAR subunits (GluA1-4), the RNA-editing proteins ADAR1-3 and possible regulators or ADAR function (PIN1, WWP2, FXR1, and CREB1). We found that the J20 mice model of AD, at a time-point exhibiting spatial memory deficits, exhibits an upregulation of the AMPAR subunit GluA3 and RNA editing enzyme ADAR1 p110 as well as a downregulation of the RNA editing enzyme ADAR2, but no change in GluA1, GluA2, GluA4, ADAR1 p150, ADAR3, PIN1, WWP2, FXR1P, or CREB1, when compared to WT mice.

### Changes in ADAR protein expression in J20 mice mirror human Alzheimer’s disease

Our findings in the J20 AD mice generally parallel what has previously been seen in human AD tissue. Specifically, our observation of an upregulation of ADAR1 p110 protein in the hippocampus parallels that observed in one study of mRNA expression in the frontal lobe of AD patients. Meanwhile, the aforementioned study reported a downregulation of ADAR1 p110 mRNA in the temporal lobes but a significant increase in the ADAR1 p150 isoform RNA in the hippocampus (Khermesh et al., 2016).

Similarly, our observation of downregulation in ADAR2 protein in the hippocampus is consistent with decreased ADAR2 mRNA expression in the prefrontal cortex (Ma et al., 2021) and caudate nucleus (Gaisler-Salomon et al., 2014) of AD patients. It is also consistent with our recent findings of ADAR2 protein downregulation in both the J20 and 5xFAD models of AD using traditional and capillary western blotting, respectively (Wright et al., 2023). However, there are differences between what we observed in J20 mice and some human studies. Our observation of ADAR2 protein downregulation in the hippocampus is in contrast to evidence indicating an upregulation of ADAR2 mRNA in the temporal lobe in AD (Khermesh et al., 2016). Furthermore, while ADAR3 mRNA has been reported to be upregulated in both the hippocampus (Annese et al., 2018) and prefrontal cortex (Ma et al., 2021), we observed no alterations in ADAR3 protein expression in the hippocampus of our J20 mice.

The underlying causes for these differences are not clear. A limitation of the prior published studies is that they have exclusively evaluated the expression of mRNA, rather than protein. It is important to note that changes in mRNA expression are often not reflected at the protein level (Nie et al., 2006; Guo et al., 2008). This is especially relevant given that RNA editing is a post-transcriptional modification and because ADAR2 can negatively regulate the efficiency of its own protein translation through self-editing (Fu et al., 2016). Thus, the RNA may be highly expressed but the self-editing may prevent protein translation. The fact we have assessed protein rather than mRNA may therefore account for some of the differences observed between our work and prior investigations in human tissue. Alternatively, it is widely acknowledged that mouse models do not perfectly replicate human AD. Specifically, all prior investigations of ADARs in AD have investigated changes in late-onset AD (LOAD) brains, which is not associated with familial AD mutations (Götz et al., 2009; Morris et al., 2014; Cho et al., 2022). Our mouse model, in contrast, is engineered to express mutations associated with familial AD and therefore may not necessarily be an optimal representation of the molecular events transpiring in LOAD.

It remains unclear if the previously published regional variability of ADAR2 mRNA within the brain also reflects a regional variation in ADAR protein levels and activity, given the complex regulation of ADARs. Nonetheless, collectively our observations and those from previous studies suggest the dysregulation of ADAR expression is a clear pathological feature of AD.

### Changes in ADAR1 and 2 have different but overlapping roles in RNA editing

ADAR1 has been identified as the principal factor responsible for the majority of editing within non-coding regions and repeat elements in mRNAs. It does not exhibit the specificity that ADAR2 has for protein-recoding genes, particularly in regards to A-I editing events that alter codons such as the GluA2 Q/R site (Tan et al., 2017; Chalk et al., 2019; Costa Cruz et al., 2020). Nevertheless, both proteins possess the capability to edit numerous identical sites and are essential for the preservation of A-to-I editing in the epitranscriptome (Wong et al., 2001). This is supported by the finding that ADAR2 KO mice retain ∼10% editing at the GluA2 Q/R site, suggesting ADAR1 may be responsible for this residual editing (Higuchi et al., 2000). Additionally, this residual editing is also present in varying degrees at other Q/R sites within GluK2, Gabra3, and Cyfip2 (Higuchi et al., 2000; Horsch et al., 2011).

Accordingly and perhaps unsurprisingly, ADAR1 and ADAR2 proteins generally show different expression profiles, are regulated differently, generally edit different targets and are differentially altered in disease (Palop et al., 2003; Esposito et al., 2006; Galvan et al., 2006; Cheng et al., 2007; Wright et al., 2013; Ameen-Ali et al., 2019). ADAR2 and its associated editing is downregulated in several conditions, and this tends to be associated with neurodegeneration (Maas et al., 2001; Peng et al., 2006; Hwang et al., 2016; Srivastava et al., 2017; Moore et al., 2019, 2019; Tran et al., 2019). In contrast, the upregulation of ADAR1 appears to have both ameliorative and pathological effects (Yang et al., 2003; Hartner et al., 2009; Sagredo et al., 2020; de Santiago et al., 2021; Cai et al., 2023). While beyond the scope of this study, future research will be needed to identify if ADARs are dysregulated at earlier time points in mouse models of AD, and if this dysregulation may precede amyloid pathologies and/or whether it plays a role in initiating other AD-associated pathologies beyond loss of dendritic spines and neurodegeneration (Konen et al., 2020; Wright et al., 2023).

### GluA3 upregulation has been observed in human AD and other neurological diseases

It is noteworthy that there is a significant increase in expression of the GluA3 AMPA receptor subunit at 44-weeks in our J20 AD model mice. The result is consistent with the recent report of a significant increase in GluA3 in the CSF of AD patients when compared to cognitively normal patients with subjective cognitive decline (SCD) (Enache et al., 2020). An increase in GluA3 has also been observed in ALS, where it appears that the co-occurrence of ADAR2 downregulation and upregulation of GluA3-containing AMPARs in motor neurons is associated with excitotoxicity and neuronal death (Rembach et al., 2004; Kwak et al., 2010; Gregory et al., 2020). Our findings therefore appear consistent with prior literature.

Interestingly, in contrast, the 3xTg-AD mouse model of AD shows an increase in the expression of the GluA3 subunit at 3 months of age, transitioning to a profound decrease at 12 months of age (Cantanelli et al., 2014). It is possible the 3xTg-AD model may have a different temporal profile of GluA3 expression compared with the J20 model, due to a different pathological profile, e.g., the added presence of neurofibrillary tau tangles (Oddo et al., 2003). Alternatively, the decrease in GluA3 expression may occur at a later time point in J20 mice. It would be useful in future work to determine if the expression of GluA3 declines in J20 mice beyond 44-weeks of age.

In order to consider why GluA3 is upregulated in AD, it is instructive to consider the role of GluA3 at the synapse. GluA3-containing AMPARs are enriched at the synapse when compared to other subunit combinations (Jacob and Weinberg, 2015) and despite their strong presence and preferential placement at synaptic junctions, their contribution to synaptic currents is minimal (Shi et al., 2001, 2001). However, the GluA3 subunit is required for beta-amyloid (Aβ)-mediated synaptic and cognitive deficits (Reinders et al., 2016). Remarkably, hippocampal neurons that do not express GluA3 are protected against Aβ-mediated synaptic depression, spine loss, and degeneration.

Based on the above, we speculate that the initial upregulation in the expression of GluA3 might serve to reduce calcium influx and/or neuronal excitability as a neuroprotective mechanism since AMPA receptors (AMPARs) composed of GluA2/3 subunits show reduced conductance compared to those containing GluA1/2 subunits (Jacob and Weinberg, 2015; Renner et al., 2017). It may then be the case that these neurons become more vulnerable to Aβ-induced toxicity at a later stage, given, as above, GluA3 neurons are more susceptible to Aβ mediated toxicity.

### The relationships between AMPA receptor subunits and editing related protein changes

We observed a positive correlation between GluA3 and ADAR2 expression across individual mice. If this were to hold up to further investigation, this finding appears at odds with our observation that, on average, GluA3 was increased and ADAR2 was decreased in J20 mice compared to controls. This provides a hint that the regulation of the expression of these two proteins may be interrelated in a complex manner.

The expression of several proteins known to bind to and regulate ADARs (PIN1, WWP2, FXR1P, and CREB1) were unaltered in the hippocampus of J20 mice. It nevertheless remains possible that these proteins influence the expression and activity of ADARs, especially since protein expression is only one of many determinants of a protein’s activity.

Interestingly, PIN1 and CREB1 exhibited inverse relationships with specific AMPAR subunits in specific mice. Specifically, in the J20 mice model, PIN1 expression was consistently negatively correlated with GluA1, GluA3, and GluA4. Although the direct role of PIN1 in moderating AMPAR expression and function is not documented, one study found that PIN1 ablation in mice induced GluA1 phosphorylation and epileptic seizures (Hou et al., 2021). Moreover, PIN1’s ability to regulate ADAR2 nuclear localisation and facilitate editing of GluA2 offers a plausible theoretical pathway linking these proteins (Marcucci et al., 2011; Behm et al., 2017). In view of that, it is interesting that GluA2 expression was not correlated with PIN1, in either genotype. As above, other changes in PIN1, such as changes in phosphorylation, may modify PIN1 interactions and regulation of GluA2.

Perhaps less surprisingly, the CREB1 protein displayed a positive correlation with the expression of GluA1, GluA2, and GluA3. A substantial body of literature underscores the role of CREB1 in regulating AMPAR subunits. Consistent with our findings, GluA1 appears to have a significant relationship with CREB signalling. Genomic analysis has revealed the potential of CREB1 to bind to at least four identified CRE sequences within the GluA1 promoter (Borges and Dingledine, 2001; Carlezon et al., 2005). Further investigations into the role of CREB1 in synaptic plasticity and learning have shown that inhibition of CREB1 function not only led to a significant reduction in the GluA1 subunit but also potentially has a downregulatory effect on GluA2 and GluA3 subunits as well (Middei et al., 2013). Moreover, it has been hypothesised that CREB1 may act to induce GluA2 expression through the well-established FosB pathway, which has been strongly implicated in AD pathology in both mice and humans (Carlezon et al., 1998; Kelz et al., 1999; McClung and Nestler, 2003). These findings suggest intriguing potential signalling relationships that may play a role in regulating both RNA-editing and AMPAR function in the J20 mice.

### Limitations and conclusion

#### Spatial

In the present study, expression profiles of the proteins of interest were derived from whole hippocampal total protein. There is, however, evidence of complex differential expression in ADAR and GluA subunits across brain regions (Jacobs et al., 2009; Schwenk et al., 2014), subregions of the hippocampus (Gorter et al., 1997; Brande-Eilat et al., 2015), and even in specific cell populations (Gal-Mark et al., 2017; Ceprian and Fulton, 2019; Cuddleston et al., 2022). In future, a more specific spatial and cell-specific study will be of value. For example, it would be interesting to use a highly sensitive method like automated capillary western to investigate the subregions of the hippocampus, specifically CA1, CA3, and DG, to understand how the progressing pathology of AD (Padurariu et al., 2012; Ugolini et al., 2018; Shipton et al., 2022) is related to RNA-editing and ADAR expression. Beyond that, a brain-wide atlas of RNA editing changes at regional and cellular levels would be highly interesting.

#### Temporal

Considering our study was restricted to one age (44-weeks), expanding our research to earlier and later stages and combining this with other behavioural/histopathological analyses in future work will allow for a better understanding of how changes in these proteins link to the development of other well-documented J20 pathologies, such as neurodegeneration, glial activation, Aβ accumulation, and dendritic spine loss (Wright et al., 2013; Ameen-Ali et al., 2019). Future research will be needed to identify if ADAR dysregulation at earlier time points may precede amyloid pathology.

#### Mouse models and gender

It would be worth investigating if our findings are consistent across sexes, and in other models of AD, particularly those that may provide a better representation of LOAD (Götz et al., 2009; Sanchez-Varo et al., 2022; Sasaguri et al., 2022) and/or that include the influence of environmental risk factors for AD such as high glucose diets, traumatic brain injury, and sleep deprivation (Zhang et al., 2021).

#### Biological complexity

While our study has identified some significant changes in protein expression and interesting protein-protein correlations, this is not representative or fully accurate of all the complex changes that may be occurring. A range of RNA and protein modifications profoundly alter protein function. Further factors may influence RNA editing and calcium permeability outside of ADAR or GluA expression, including the mislocalisation of ADARs (specifically ADAR1 p150), alterations in mRNA-ADAR binding affinity or structure, changes in the ratio of AMPAR subunit conformation and trafficking of AMPAR subunits to the synapse.

#### Methodological limitations

Finally, even with a highly sensitive tool like capillary western blotting, it is still difficult to quantify lowly expressed proteins such as ADAR1 p150 and ADAR3, leading to a high level of variance in the data. Unfortunately, there are few accessible tools to address this issue at present.

## Conclusion

In summary, the dysregulation of ADAR and GluA proteins in J20 mice show significant parallels with pathological changes observed in human AD. Further investigation is required to determine whether changes in ADAR expression lead to functional alterations at RNA editing sites, particularly the GluA2 Q/R site. Furthermore, the role that these dysregulated proteins have in the development of well-established AD-related pathologies, such as tau hyperphosphorylation, neurofibrillary tangles, Aβ plaques, the immune response, and memory deficits, is still unclear.

## Data availability statement

The original contributions presented in the study are included in the article/Supplementary material, further inquiries can be directed to the corresponding author.

## Ethics statement

The animal study was approved by the Garvan Institute and St. Vincent’s Hospital Animal Ethics Committee. The study was conducted in accordance with the local legislation and institutional requirements.

## Author contributions

LM: Writing – original draft, Writing – review and editing, Data curation, Formal Analysis, Investigation, Validation, Visualization. GM: Writing – original draft, Writing – review and editing, Conceptualization, Formal Analysis, Methodology, Supervision, Validation, Visualization. LK: Writing – review and editing, Conceptualization, Methodology, Supervision. PR: Writing – review and editing, Formal Analysis, Investigation, Validation, Visualization. NA: Writing – review and editing, Formal Analysis, Visualization. BV: Writing – review and editing, Conceptualization, Funding acquisition, Methodology, Project administration, Resources, Supervision.

## References

[B1] Akbarian S. Smith M. A. Jones E. G. (1995). Editing for an AMPA receptor subunit RNA in prefrontal cortex and striatum in Alzheimer’s disease, Huntington’s disease and schizophrenia. *Brain Res.* 699 297–304. 10.1016/0006-8993(95)00922-D 8616634

[B2] Aldridge G. M. Podrebarac D. M. Greenough W. T. Weiler I. J. (2008). The use of total protein stains as loading controls: An alternative to high-abundance single-protein controls in semi-quantitative immunoblotting. *J. Neurosci. Methods* 172 250–254. 10.1016/j.jneumeth.2008.05.003 18571732 PMC2567873

[B3] Ameen-Ali K. E. Simpson J. E. Wharton S. B. Heath P. R. Sharp P. S. Brezzo G. (2019). The time course of recognition memory impairment and glial pathology in the hAPP-J20 mouse model of Alzheimer’s Disease. *J. Alzheimers Dis.* 68 609–624. 10.3233/JAD-181238 30814360

[B4] Annese A. Manzari C. Lionetti C. Picardi E. Horner D. S. Chiara M. (2018). Whole transcriptome profiling of Late-Onset Alzheimer’s Disease patients provides insights into the molecular changes involved in the disease. *Sci. Rep.* 8:4282. 10.1038/s41598-018-22701-2 29523845 PMC5844946

[B5] Behm M. Wahlstedt H. Widmark A. Eriksson M. Öhman M. (2017). Accumulation of nuclear ADAR2 regulates adenosine-to-inosine RNA editing during neuronal development. *J. Cell Sci.* 130 745–753. 10.1242/jcs.200055 28082424

[B6] Borges K. Dingledine R. (2001). Functional organization of the GluR1 glutamate receptor promoter. *J. Biol. Chem.* 276 25929–25938. 10.1074/jbc.M009105200 11340067

[B7] Brande-Eilat N. Golumbic Y. N. Zaidan H. Gaisler-Salomon I. (2015). Acquisition of conditioned fear is followed by region-specific changes in RNA editing of glutamate receptors. *Stress* 18 309–318. 10.3109/10253890.2015.1073254 26383032

[B8] Brusa R. Zimmermann F. Koh D. S. Feldmeyer D. Gass P. Seeburg P. H. (1995). Early-onset epilepsy and postnatal lethality associated with an editing-deficient GluR-B allele in mice. *Science* 270 1677–1680. 10.1126/science.270.5242.1677 7502080

[B9] Bukke V. N. Archana M. Villani R. Romano A. D. Wawrzyniak A. Balawender K. (2020). The Dual Role of Glutamatergic Neurotransmission in Alzheimer’s Disease: From Pathophysiology to Pharmacotherapy. *Int. J. Mol. Sci.* 21:7452. 10.3390/ijms21207452 33050345 PMC7589203

[B10] Cai D. Fraunfelder M. Fujise K. Chen S.-Y. (2023). ADAR1 exacerbates ischemic brain injury via astrocyte-mediated neuron apoptosis. *Redox Biol*. 67:102903. 10.1016/j.redox.2023.102903 37801857 PMC10570147

[B11] Cantanelli P. Sperduti S. Ciavardelli D. Stuppia L. Gatta V. Sensi S. L. (2014). Age-dependent modifications of AMPA receptor subunit expression levels and related cognitive effects in 3xTg-AD Mice. *Front. Aging Neurosci.* 6:200. 10.3389/fnagi.2014.00200 25140151 PMC4122177

[B12] Carlezon W. A. Duman R. S. Nestler E. J. (2005). The many faces of CREB. *Trends Neurosci.* 28 436–445. 10.1016/j.tins.2005.06.005 15982754

[B13] Carlezon W. A. Thome J. Olson V. G. Lane-Ladd S. B. Brodkin E. S. Hiroi N. (1998). Regulation of cocaine reward by CREB. *Science* 282 2272–2275. 10.1126/science.282.5397.2272 9856954

[B14] Carter T. L. Rissman R. A. Mishizen-Eberz A. J. Wolfe B. B. Hamilton R. L. Gandy S. (2004). Differential preservation of AMPA receptor subunits in the hippocampi of Alzheimer’s disease patients according to Braak stage. *Exp. Neurol.* 187 299–309. 10.1016/j.expneurol.2003.12.010 15144856

[B15] Castle A. R. Daude N. Gilch S. Westaway D. (2019). Application of high-throughput, capillary-based Western analysis to modulated cleavage of the cellular prion protein. *J. Biol. Chem.* 294 2642–2650. 10.1074/jbc.RA118.006367 30578300 PMC6393610

[B16] Ceprian M. Fulton D. (2019). Glial cell AMPA receptors in nervous system health, injury and disease. *Int. J. Mol. Sci.* 20:2450. 10.3390/ijms20102450 31108947 PMC6566241

[B17] Chalk A. M. Taylor S. Heraud-Farlow J. E. Walkley C. R. (2019). The majority of A-to-I RNA editing is not required for mammalian homeostasis. *Genome Biol.* 20:268. 10.1186/s13059-019-1873-2 31815657 PMC6900863

[B18] Chaytow H. Sethw Hassan I. Akbar S. Popplewell L. Dickson G. Chen P. E. (2021). A new strategy to increase RNA editing at the Q/R site of GluA2 AMPA receptor subunits by targeting alternative splicing patterns of ADAR2. *J. Neurosci. Methods* 364:109357. 10.1016/j.jneumeth.2021.109357 34536489 PMC8573265

[B19] Cheng I. H. Scearce-Levie K. Legleiter J. Palop J. J. Gerstein H. Bien-Ly N. (2007). Accelerating amyloid-beta fibrillization reduces oligomer levels and functional deficits in Alzheimer disease mouse models. *J. Biol. Chem.* 282 23818–23828. 10.1074/jbc.M701078200 17548355

[B20] Cho J. D. Yang M. Santa-Maria I. (2022). Modeling Alzheimer’s disease: Considerations for a better translational and replicable mouse model. *Neural Regen. Res.* 17 2448–2449. 10.4103/1673-5374.335787 35535894 PMC9120700

[B21] Christofi T. Zaravinos A. (2019). RNA editing in the forefront of epitranscriptomics and human health. *J. Transl. Med.* 17:319. 10.1186/s12967-019-2071-4 31547885 PMC6757416

[B22] Cissé M. Sanchez P. E. Kim D. H. Ho K. Yu G.-Q. Mucke L. (2011). Ablation of cellular prion protein does not ameliorate abnormal neural network activity or cognitive dysfunction in the J20 line of human amyloid precursor protein transgenic mice. *J. Neurosci.* 31 10427–10431. 10.1523/JNEUROSCI.1459-11.2011 21775587 PMC3314063

[B23] Coleman P. D. Yao P. J. (2003). Synaptic slaughter in Alzheimer’s disease. *Neurobiol. Aging* 24 1023–1027. 10.1016/j.neurobiolaging.2003.09.001 14643374

[B24] Costa Cruz P. H. Kato Y. Nakahama T. Shibuya T. Kawahara Y. (2020). A comparative analysis of ADAR mutant mice reveals site-specific regulation of RNA editing. *RNA* 26 454–469. 10.1261/rna.072728.119 31941663 PMC7075269

[B25] Cuddleston W. H. Li J. Fan X. Kozenkov A. Lalli M. Khalique S. (2022). Cellular and genetic drivers of RNA editing variation in the human brain. *Nat. Commun.* 13:2997. 10.1038/s41467-022-30531-0 35637184 PMC9151768

[B26] de Santiago P. R. Blanco A. Morales F. Marcelain K. Harismendy O. Sjöberg Herrera M. (2021). Immune-related IncRNA LINC00944 responds to variations in ADAR1 levels and it is associated with breast cancer prognosis. *Life Sci.* 268:118956. 10.1016/j.lfs.2020.118956 33383047

[B27] Dhungana A. Becchi S. Leake J. Morris G. Avgan N. Balleine B. W. (2023). Goal-directed action is initially impaired in a hAPP-J20 mouse model of Alzheimer’s Disease. *eNeuro* 10 ENEURO.363–ENEURO.322. 10.1523/ENEURO.0363-22.2023 36650070 PMC9927544

[B28] Enache D. Pereira J. Jelic V. Winblad B. Nilsson P. Aarsland D. (2020). Increased cerebrospinal fluid concentration of ZnT3 is associated with cognitive impairment in Alzheimer’s disease. *J. Alzheimers Dis.* 77:200498. 10.3233/JAD-200498 32925049

[B29] Esposito L. Raber J. Kekonius L. Yan F. Yu G.-Q. Bien-Ly N. (2006). Reduction in mitochondrial superoxide dismutase modulates Alzheimer’s disease-like pathology and accelerates the onset of behavioral changes in human amyloid precursor protein transgenic mice. *J. Neurosci.* 26 5167–5179. 10.1523/JNEUROSCI.0482-06.2006 16687508 PMC6674260

[B30] Feldmeyer D. Kask K. Brusa R. Kornau H. C. Kolhekar R. Rozov A. (1999). Neurological dysfunctions in mice expressing different levels of the Q/R site-unedited AMPAR subunit GluR-B. *Nat. Neurosci.* 2 57–64. 10.1038/4561 10195181

[B31] Ferguson R. E. Carroll H. P. Harris A. Maher E. R. Selby P. J. Banks R. E. (2005). Housekeeping proteins: A preliminary study illustrating some limitations as useful references in protein expression studies. *PROTEOMICS* 5 566–571. 10.1002/pmic.200400941 15627964

[B32] Filippini A. Bonini D. Lacoux C. Pacini L. Zingariello M. Sancillo L. (2017). Absence of the Fragile X Mental retardation protein results in defects of RNA editing of neuronal mRNAs in mouse. *RNA Biol.* 14 1580–1591. 10.1080/15476286.2017.1338232 28640668 PMC5785225

[B33] Fu Y. Zhao X. Li Z. Wei J. Tian Y. (2016). Splicing variants of ADAR2 and ADAR2-mediated RNA editing in glioma. *Oncol. Lett.* 12 788–792. 10.3892/ol.2016.4734 27446352 PMC4950497

[B34] Gaisler-Salomon I. Kravitz E. Feiler Y. Safran M. Biegon A. Amariglio N. (2014). Hippocampus-specific deficiency in RNA editing of GluA2 in Alzheimer’s disease. *Neurobiol. Aging* 35 1785–1791. 10.1016/j.neurobiolaging.2014.02.018 24679603

[B35] Gal-Mark N. Shallev L. Sweetat S. Barak M. Billy, Li J. (2017). Abnormalities in A-to-I RNA editing patterns in CNS injuries correlate with dynamic changes in cell type composition. *Sci. Rep.* 7:43421. 10.1038/srep43421 28266523 PMC5339895

[B36] Galvan V. Gorostiza O. F. Banwait S. Ataie M. Logvinova A. V. Sitaraman S. (2006). Reversal of Alzheimer’s-like pathology and behavior in human APP transgenic mice by mutation of Asp664. *Proc. Natl. Acad. Sci. U. S. A.* 103 7130–7135. 10.1073/pnas.0509695103 16641106 PMC1459029

[B37] Gan Q. Dai J. Zhou H.-X. Wollmuth L. P. (2016). The Transmembrane Domain Mediates Tetramerization of α-Amino-3-hydroxy-5-methyl-4-isoxazolepropionic Acid (AMPA) Receptors. *J. Biol. Chem.* 291 6595–6606. 10.1074/jbc.M115.686246 26839312 PMC4813562

[B38] Gannon H. S. Zou T. Kiessling M. K. Gao G. F. Cai D. Choi P. S. (2018). Identification of ADAR1 adenosine deaminase dependency in a subset of cancer cells. *Nat. Commun.* 9:5450. 10.1038/s41467-018-07824-4 30575730 PMC6303303

[B39] Gardner O. K. Wang L. Van Booven D. Whitehead P. L. Hamilton-Nelson K. L. Adams L. D. (2019). RNA editing alterations in a multi-ethnic Alzheimer disease cohort converge on immune and endocytic molecular pathways. *Hum. Mol. Genet.* 28 3053–3061. 10.1093/hmg/ddz110 31162550 PMC6737295

[B40] Gassner F. J. Zaborsky N. Buchumenski I. Levanon E. Y. Gatterbauer M. Schubert M. (2021). RNA editing contributes to epitranscriptome diversity in chronic lymphocytic leukemia. *Leukemia* 35 1053–1063. 10.1038/s41375-020-0995-6 32728184 PMC8024191

[B41] George C. X. Ramaswami G. Li J. B. Samuel C. E. (2016). Editing of Cellular Self-RNAs by Adenosine Deaminase ADAR1 Suppresses Innate Immune Stress Responses *. *J. Biol. Chem.* 291 6158–6168. 10.1074/jbc.M115.709014 26817845 PMC4813567

[B42] Gómez-Isla T. Hollister R. West H. Mui S. Growdon J. H. Petersen R. C. (1997). Neuronal loss correlates with but exceeds neurofibrillary tangles in Alzheimer’s disease. *Ann. Neurol.* 41 17–24. 10.1002/ana.410410106 9005861

[B43] Gong C.-X. Liu F. Iqbal K. (2018). Multifactorial hypothesis and multi-targets for Alzheimer’s disease. *J. Alzheimers Dis.* 64 S107–S117. 10.3233/JAD-179921 29562523

[B44] Gorter J. A. Petrozzino J. J. Aronica E. M. Rosenbaum D. M. Opitz T. Bennett M. V. (1997). Global ischemia induces downregulation of Glur2 mRNA and increases AMPA receptor-mediated Ca2+ influx in hippocampal CA1 neurons of gerbil. *J. Neurosci.* 17 6179–6188. 10.1523/JNEUROSCI.17-16-06179.1997 9236229 PMC6568367

[B45] Götz J. Schonrock N. Vissel B. Ittner L. M. (2009). Alzheimer’s disease selective vulnerability and modeling in transgenic mice. *J. Alzheimers Dis.* 18 243–251. 10.3233/JAD-2009-1143 19584440

[B46] Greenamyre J. T. Maragos W. F. Albin R. L. Penney J. B. Young A. B. (1988). Glutamate transmission and toxicity in Alzheimer’s disease. *Prog. Neuropsychopharmacol. Biol. Psychiatry* 12 421–430. 10.1016/0278-5846(88)90102-9 2900537

[B47] Gregory J. M. Livesey M. R. McDade K. Selvaraj B. T. Barton S. K. Chandran S. (2020). Dysregulation of AMPA receptor subunit expression in sporadic ALS post-mortem brain. *J. Pathol.* 250 67–78. 10.1002/path.5351 31579943 PMC6973025

[B48] Guo X. Wiley C. A. Steinman R. A. Sheng Y. Ji B. Wang J. (2021). Aicardi-Goutières syndrome-associated mutation at ADAR1 gene locus activates innate immune response in mouse brain. *J. Neuroinflammation* 18:169. 10.1186/s12974-021-02217-9 34332594 PMC8325854

[B49] Guo Y. Xiao P. Lei S. Deng F. Xiao G. G. Liu Y. (2008). How is mRNA expression predictive for protein expression? A correlation study on human circulating monocytes. *Acta Biochim. Biophys. Sin.* 40 426–436. 10.1111/j.1745-7270.2008.00418.x 18465028

[B50] Harris J. A. Devidze N. Halabisky B. Lo I. Thwin M. T. Yu G.-Q. (2010). Many neuronal and behavioral impairments in transgenic mouse models of Alzheimer’s disease are independent of caspase cleavage of the amyloid precursor protein. *J. Neurosci.* 30 372–381. 10.1523/JNEUROSCI.5341-09.2010 20053918 PMC3064502

[B51] Hartner J. C. Walkley C. R. Lu J. Orkin S. H. (2009). ADAR1 is essential for the maintenance of hematopoiesis and suppression of interferon signaling. *Nat. Immunol.* 10, 109–115. 10.1038/ni.1680 19060901 PMC2701568

[B52] Helm M. Motorin Y. (2017). Detecting RNA modifications in the epitranscriptome: predict and validate. *Nat. Rev. Genet.* 18 275–291. 10.1038/nrg.2016.169 28216634

[B53] Higuchi M. Maas S. Single F. N. Hartner J. Rozov A. Burnashev N. (2000). Point mutation in an AMPA receptor gene rescues lethality in mice deficient in the RNA-editing enzyme ADAR2. *Nature* 406 78–81. 10.1038/35017558 10894545

[B54] Hollmann M. Hartley M. Heinemann S. (1991). Ca2+ permeability of KA-AMPA–gated glutamate receptor channels depends on subunit composition. *Science* 252 851–853. 10.1126/science.1709304 1709304

[B55] Honer W. G. Dickson D. W. Gleeson J. Davies P. (1992). Regional synaptic pathology in Alzheimer’s disease. *Neurobiol. Aging* 13 375–382. 10.1016/0197-4580(92)90111-A 1625766

[B56] Horsch M. Seeburg P. H. Adler T. Aguilar-Pimentel J. A. Becker L. Calzada-Wack J. (2011). Requirement of the RNA-editing Enzyme ADAR2 for Normal Physiology in Mice. *J. Biol. Chem.* 286 18614–18622. 10.1074/jbc.M110.200881 21467037 PMC3099677

[B57] Hou X. Yang F. Li A. Zhao D. Ma N. Chen L. (2021). The Pin1-CaMKII-AMPA receptor axis regulates epileptic susceptibility. *Cereb. Cortex* 31 3082–3095. 10.1093/cercor/bhab004 33569579 PMC8107790

[B58] Hume R. I. Dingledine R. Heinemann S. F. (1991). Identification of a site in glutamate receptor subunits that controls calcium permeability. *Science* 253 1028–1031. 10.1126/science.1653450 1653450

[B59] Hwang T. Park C.-K. Leung A. K. L. Gao Y. Hyde T. M. Kleinman J. E. (2016). Dynamic regulation of RNA editing in human brain development and disease. *Nat. Neurosci.* 19 1093–1099. 10.1038/nn.4337 27348216

[B60] Hyman B. T. Penney J. B. Blackstone C. D. Young A. B. (1994). Localization of non-N-methyl-D-aspartate glutamate receptors in normal and Alzheimer hippocampal formation. *Ann. Neurol.* 35 31–37. 10.1002/ana.410350106 8285589

[B61] Hynd M. R. Scott H. L. Dodd P. R. (2004). Glutamate-mediated excitotoxicity and neurodegeneration in Alzheimer’s disease. *Neurochem. Int.* 45 583–595. 10.1016/j.neuint.2004.03.007 15234100

[B62] Isaac J. T. R. Ashby M. C. McBain C. J. (2007). The Role of the GluR2 subunit in AMPA receptor function and synaptic plasticity. *Neuron* 54 859–871. 10.1016/j.neuron.2007.06.001 17582328

[B63] Italia M. Ferrari E. Di Luca M. Gardoni F. (2021). GluA3-containing AMPA receptors: From physiology to synaptic dysfunction in brain disorders. *Neurobiol. Dis.* 161:105539. 10.1016/j.nbd.2021.105539 34743951

[B64] Jacob A. L. Weinberg R. J. (2015). The organization of AMPA receptor subunits at the postsynaptic membrane. *Hippocampus* 25 798–812. 10.1002/hipo.22404 25524891 PMC4472633

[B65] Jacobs M. M. Fogg R. L. Emeson R. B. Stanwood G. D. (2009). ADAR1 and ADAR2 expression and editing activity during forebrain development. *DNE* 31 223–237. 10.1159/000210185 19325227 PMC2692045

[B66] Kelz M. B. Chen J. Carlezon W. A. Whisler K. Gilden L. Beckmann A. M. (1999). Expression of the transcription factor deltaFosB in the brain controls sensitivity to cocaine. *Nature* 401 272–276. 10.1038/45790 10499584

[B67] Khermesh K. D’Erchia A. M. Barak M. Annese A. Wachtel C. Levanon E. Y. (2016). Reduced levels of protein recoding by A-to-I RNA editing in Alzheimer’s disease. *RNA* 22 290–302. 10.1261/rna.054627.115 26655226 PMC4712678

[B68] Kirshner Z. Z. Gibbs R. B. (2018). Use of the REVERT^®^ total protein stain as a loading control demonstrates significant benefits over the use of housekeeping proteins when analyzing brain homogenates by Western blot: An analysis of samples representing different gonadal hormone states. *Mol. Cell. Endocrinol.* 473 156–165. 10.1016/j.mce.2018.01.015 29396126 PMC6045444

[B69] Koller A. Wätzig H. (2005). Precision and variance components in quantitative gel electrophoresis. *Electrophoresis* 26 2470–2475. 10.1002/elps.200500024 15924365

[B70] Konen L. M. Wright A. L. Royle G. A. Morris G. P. Lau B. K. Seow P. W. (2020). A new mouse line with reduced GluA2 Q/R site RNA editing exhibits loss of dendritic spines, hippocampal CA1-neuron loss, learning and memory impairments and NMDA receptor-independent seizure vulnerability. *Mol. Brain* 13:27. 10.1186/s13041-020-0545-1 32102661 PMC7045468

[B71] Kurup R. R. Oakes E. K. Manning A. C. Mukherjee P. Vadlamani P. Hundley H. A. (2022). RNA binding by ADAR3 inhibits adenosine-to-inosine editing and promotes expression of immune response protein MAVS. *J. Biol. Chem.* 298:102267. 10.1016/j.jbc.2022.102267 35850307 PMC9418441

[B72] Kwak S. Hideyama T. Yamashita T. Aizawa H. (2010). AMPA receptor-mediated neuronal death in sporadic ALS. *Neuropathology* 30 182–188. 10.1111/j.1440-1789.2009.01090.x 20102521

[B73] Li R. Shen Y. (2013). An old method facing a new challenge: re-visiting housekeeping proteins as internal reference control for neuroscience research. *Life Sci.* 92 747–751. 10.1016/j.lfs.2013.02.014 23454168 PMC3614345

[B74] Li T. Yang X. Li W. Song J. Li Z. Zhu X. (2021). ADAR1 Stimulation by IFN-α Downregulates the Expression of MAVS via RNA Editing to Regulate the Anti-HBV Response. *Mol. Therapy* 29 1335–1348. 10.1016/j.ymthe.2020.11.031 33279720 PMC7934633

[B75] Lin J. Redies C. (2012). Histological evidence: housekeeping genes beta-actin and GAPDH are of limited value for normalization of gene expression. *Dev. Genes Evol.* 222 369–376. 10.1007/s00427-012-0420-x 23099774

[B76] Liu Y. Samuel C. E. (1999). Editing of Glutamate Receptor Subunit B Pre-mRNA by Splice-site Variants of Interferon-inducible Double-stranded RNA-specific Adenosine Deaminase ADAR1*. *J. Biol. Chem.* 274 5070–5077. 10.1074/jbc.274.8.5070 9988754

[B77] Ma Y. Dammer E. B. Felsky D. Duong D. M. Klein H.-U. White C. C. (2021). Atlas of RNA editing events affecting protein expression in aged and Alzheimer’s disease human brain tissue. *Nat. Commun.* 12:7035. 10.1038/s41467-021-27204-9 34857756 PMC8640037

[B78] Maas S. Kawahara Y. Tamburro K. M. Nishikura K. (2006). A-to-I RNA editing and human disease. *RNA Biol.* 3 1–9. 10.4161/rna.3.1.2495 17114938 PMC2947206

[B79] Maas S. Patt S. Schrey M. Rich A. (2001). Underediting of glutamate receptor GluR-B mRNA in malignant gliomas. *Proc. Natl. Acad. Sci. U. S. A.* 98 14687–14692. 10.1073/pnas.251531398 11717408 PMC64742

[B80] Mannion N. M. Greenwood S. M. Young R. Cox S. Brindle J. Read D. (2014). The RNA-editing enzyme ADAR1 controls innate immune responses to RNA. *Cell Rep.* 9 1482–1494. 10.1016/j.celrep.2014.10.041 25456137 PMC4542304

[B81] Marcucci R. Brindle J. Paro S. Casadio A. Hempel S. Morrice N. (2011). Pin1 and WWP2 regulate GluR2 Q/R site RNA editing by ADAR2 with opposing effects. *EMBO J.* 30 4211–4222. 10.1038/emboj.2011.303 21847096 PMC3199391

[B82] McClung C. A. Nestler E. J. (2003). Regulation of gene expression and cocaine reward by CREB and DeltaFosB. *Nat. Neurosci.* 6 1208–1215. 10.1038/nn1143 14566342

[B83] Middei S. Houeland G. Cavallucci V. Ammassari-Teule M. D’Amelio M. Marie H. (2013). CREB is necessary for synaptic maintenance and learning-induced changes of the AMPA receptor GluA1 subunit. *Hippocampus* 23 488–499. 10.1002/hipo.22108 23504989

[B84] Mladenova D. Barry G. Konen L. M. Pineda S. S. Guennewig B. Avesson L. (2018). Adar3 is involved in learning and memory in mice. *Front. Neurosci.* 12:243. 10.3389/fnins.2018.00243 29719497 PMC5914295

[B85] Moore S. Alsop E. Lorenzini I. Starr A. Rabichow B. E. Mendez E. (2019). ADAR2 Mislocalization and Widespread RNA Editing Aberrations in C9orf72-Mediated ALS/FTD. *Acta Neuropathol.* 138 49–65. 10.1007/s00401-019-01999-w 30945056 PMC6750285

[B86] Morris G. P. Clark I. A. Vissel B. (2014). Inconsistencies and controversies surrounding the amyloid hypothesis of Alzheimer’s disease. *Acta Neuropathol. Commun.* 2:135. 10.1186/s40478-014-0135-5 25231068 PMC4207354

[B87] Morris G. P. Clark I. A. Vissel B. (2018). Questions concerning the role of amyloid-β in the definition, aetiology and diagnosis of Alzheimer’s disease. *Acta Neuropathol.* 136 663–689. 10.1007/s00401-018-1918-8 30349969 PMC6208728

[B88] Nemlich Y. Greenberg E. Ortenberg R. Besser M. J. Barshack I. Jacob-Hirsch J. (2013). MicroRNA-mediated loss of ADAR1 in metastatic melanoma promotes tumor growth. *J. Clin. Invest.* 123 2703–2718. 10.1172/JCI62980 23728176 PMC3668823

[B89] Nie L. Wu G. Zhang W. (2006). Correlation of mRNA expression and protein abundance affected by multiple sequence features related to translational efficiency in desulfovibrio vulgaris: A quantitative analysis. *Genetics* 174 2229–2243. 10.1534/genetics.106.065862 17028312 PMC1698625

[B90] Nixon R. A. (2017). Amyloid precursor protein and endosomal–lysosomal dysfunction in Alzheimer’s disease: inseparable partners in a multifactorial disease. *FASEB J.* 31 2729–2743. 10.1096/fj.201700359 28663518 PMC6137496

[B91] Oddo S. Caccamo A. Shepherd J. D. Murphy M. P. Golde T. E. Kayed R. (2003). Triple-transgenic model of Alzheimer’s disease with plaques and tangles: Intracellular Aβ and synaptic dysfunction. *Neuron* 39 409–421. 10.1016/S0896-6273(03)00434-3 12895417

[B92] Pachernegg S. Münster Y. Muth-Köhne E. Fuhrmann G. Hollmann M. (2015). GluA2 is rapidly edited at the Q/R site during neural differentiation in vitro. *Front. Cell. Neurosci.* 9:69. 10.3389/fncel.2015.00069 25798088 PMC4350408

[B93] Padurariu M. Ciobica A. Mavroudis I. Fotiou D. Baloyannis S. (2012). Hippocampal neuronal loss in the CA1 and CA3 areas of Alzheimer’s disease patients. *Psychiatr. Danub.* 24 152–158. 22706413

[B94] Palop J. J. Jones B. Kekonius L. Chin J. Yu G.-Q. Raber J. (2003). Neuronal depletion of calcium-dependent proteins in the dentate gyrus is tightly linked to Alzheimer’s disease-related cognitive deficits. *Proc. Natl. Acad. Sci. U. S. A.* 100 9572–9577. 10.1073/pnas.1133381100 12881482 PMC170959

[B95] Peng P. L. Zhong X. Tu W. Soundarapandian M. M. Molner P. Zhu D. (2006). ADAR2-Dependent RNA Editing of AMPA Receptor Subunit GluR2 Determines Vulnerability of Neurons in Forebrain Ischemia. *Neuron* 49 719–733. 10.1016/j.neuron.2006.01.025 16504947

[B96] Pláteník J. Fišar Z. Buchal R. Jirák R. Kitzlerová E. Zvěřová M. (2014). GSK3β, CREB, and BDNF in peripheral blood of patients with Alzheimer’s disease and depression. *Prog. Neuro-Psychopharmacol. Biol. Psychiatry* 50 83–93. 10.1016/j.pnpbp.2013.12.001 24334212

[B97] Ramírez-Moya J. Baker A. R. Slack F. J. Santisteban P. (2020). ADAR1-mediated RNA editing is a novel oncogenic process in thyroid cancer and regulates miR-200 activity. *Oncogene* 39 3738–3753. 10.1038/s41388-020-1248-x 32157211 PMC7190574

[B98] Reinders N. R. Pao Y. Renner M. C. da Silva-Matos C. M. Lodder T. R. Malinow R. (2016). Amyloid-β effects on synapses and memory require AMPA receptor subunit GluA3. *Proc. Natl. Acad. Sci.* 113 E6526–E6534. 10.1073/pnas.1614249113 27708157 PMC5081598

[B99] Rembach A. Turner B. J. Bruce S. Cheah I. K. Scott R. L. Lopes E. C. (2004). Antisense peptide nucleic acid targeting GluR3 delays disease onset and progression in the SOD1 G93A mouse model of familial ALS. *J. Neurosci. Res.* 77 573–582. 10.1002/jnr.20191 15264227

[B100] Renner M. C. Albers E. H. Gutierrez-Castellanos N. Reinders N. R. van Huijstee A. N. Xiong H. (2017). Synaptic plasticity through activation of GluA3-containing AMPA-receptors. *Elife* 6 e25462. 10.7554/eLife.25462 28762944 PMC5578739

[B101] Rossmann M. Sukumaran M. Penn A. C. Veprintsev D. B. Babu M. M. Greger I. H. (2011). Subunit-selective N-terminal domain associations organize the formation of AMPA receptor heteromers. *EMBO J.* 30 959–971. 10.1038/emboj.2011.16 21317873 PMC3049212

[B102] Rustandi R. R. Loughney J. W. Hamm M. Hamm C. Lancaster C. Mach A. (2012). Qualitative and quantitative evaluation of Simon™, a new CE-based automated Western blot system as applied to vaccine development. *Electrophoresis* 33 2790–2797. 10.1002/elps.201200095 22965727

[B103] Sagredo E. A. Sagredo A. I. Blanco A. Rojas De Santiago P. Rivas S. Assar R. (2020). ADAR1 transcriptome editing promotes breast cancer progression through the regulation of cell cycle and DNA damage response. *Biochim. Biophys. Acta Mol. Cell Res*. 1867:118716. 10.1016/j.bbamcr.2020.118716 32275931

[B104] Saletore Y. Meyer K. Korlach J. Vilfan I. D. Jaffrey S. Mason C. E. (2012). The birth of the Epitranscriptome: deciphering the function of RNA modifications. *Genome Biol.* 13:175. 10.1186/gb-2012-13-10-175 23113984 PMC3491402

[B105] Sanchez-Varo R. Mejias-Ortega M. Fernandez-Valenzuela J. J. Nuñez-Diaz C. Caceres-Palomo L. Vegas-Gomez L. (2022). Transgenic Mouse Models of Alzheimer’s Disease: An Integrative Analysis. *Int. J. Mol. Sc.i* 23:5404. 10.3390/ijms23105404 35628216 PMC9142061

[B106] Sasaguri H. Hashimoto S. Watamura N. Sato K. Takamura R. Nagata K. (2022). Recent Advances in the Modeling of Alzheimer’s Disease. *Front. Neurosci.* 16:807473. 10.3389/fnins.2022.807473 35431779 PMC9009508

[B107] Savva Y. A. Rieder L. E. Reenan R. A. (2012). The ADAR protein family. *Genome Biol.* 13:252. 10.1186/gb-2012-13-12-252 23273215 PMC3580408

[B108] Scheff S. W. Neltner J. H. Nelson P. T. (2014). Is synaptic loss a unique hallmark of Alzheimer’s disease? *Biochem. Pharmacol.* 88 517–528. 10.1016/j.bcp.2013.12.028 24412275 PMC4230706

[B109] Schwenk J. Baehrens D. Haupt A. Bildl W. Boudkkazi S. Roeper J. (2014). Regional Diversity and Developmental Dynamics of the AMPA-Receptor Proteome in the Mammalian Brain. *Neuron* 84 41–54. 10.1016/j.neuron.2014.08.044 25242221

[B110] Sebe J. Y. Cho S. Sheets L. Rutherford M. A. Gersdorff H. von (2017). Ca2+-Permeable AMPARs Mediate Glutamatergic Transmission and Excitotoxic Damage at the Hair Cell Ribbon Synapse. *J. Neurosci.* 37 6162–6175. 10.1523/JNEUROSCI.3644-16.2017 28539424 PMC5481947

[B111] Shi S. Hayashi Y. Esteban J. A. Malinow R. (2001). Subunit-specific rules governing AMPA receptor trafficking to synapses in hippocampal pyramidal neurons. *Cell* 105 331–343. 10.1016/s0092-8674(01)00321-x 11348590

[B112] Shipton O. A. Tang C. S. Paulsen O. Vargas-Caballero M. (2022). Differential vulnerability of hippocampal CA3-CA1 synapses to Aβ. *Acta Neuropathol. Commun.* 10:45. 10.1186/s40478-022-01350-7 35379353 PMC8981624

[B113] Sommer B. Köhler M. Sprengel R. Seeburg P. H. (1991). RNA editing in brain controls a determinant of ion flow in glutamate-gated channels. *Cell* 67 11–19. 10.1016/0092-8674(91)90568-J 1717158

[B114] Song Z. Ding Q. Yang Y. (2023). Orchestration of a blood-derived and ADARB1-centred network in Alzheimer’s and Parkinson’s disease. *Cell. Signal.* 110:110845. 10.1016/j.cellsig.2023.110845 37544632

[B115] Sormunen A. Koivulehto E. Alitalo K. Saksela K. Laham-Karam N. Ylä-Herttuala S. (2023). Comparison of automated and traditional western blotting methods. *Methods Protoc.* 6:43. 10.3390/mps6020043 37104025 PMC10142486

[B116] Srivastava P. K. Bagnati M. Delahaye-Duriez A. Ko J.-H. Rotival M. Langley S. R. (2017). Genome-wide analysis of differential RNA editing in epilepsy. *Genome Res.* 27 440–450. 10.1101/gr.210740.116 28250018 PMC5340971

[B117] Takuma H. Kwak S. Yoshizawa T. Kanazawa I. (1999). Reduction of GluR2 RNA editing, a molecular change that increases calcium influx through AMPA receptors, selective in the spinal ventral gray of patients with amyotrophic lateral sclerosis. *Ann. Neurol.* 46 806–815. 10.1002/1531-8249(199912)46:6<806::aid-ana2>3.0.co;2-s 10589532

[B118] Tan M. H. Li Q. Shanmugam R. Piskol R. Kohler J. Young A. N. (2017). Dynamic landscape and regulation of RNA editing in mammals. *Nature* 550 249–254. 10.1038/nature24041 29022589 PMC5723435

[B119] Tran S. S. Jun H.-I. Bahn J. H. Azghadi A. Ramaswami G. Van Nostrand E. L. (2019). Widespread RNA editing dysregulation in brains from autistic individuals. *Nat. Neurosci.* 22 25–36. 10.1038/s41593-018-0287-x 30559470 PMC6375307

[B120] Ugolini F. Lana D. Nardiello P. Nosi D. Pantano D. Casamenti F. (2018). Different Patterns of Neurodegeneration and Glia Activation in CA1 and CA3 Hippocampal Regions of TgCRND8 Mice. *Front. Aging Neurosci.* 10:372. 10.3389/fnagi.2018.00372 30483118 PMC6243135

[B121] Van Damme P. Van den Bosch L. Van Houtte E. Callewaert G. Robberecht W. (2002). GluR2-dependent properties of AMPA receptors determine the selective vulnerability of motor neurons to excitotoxicity. *J. Neurophysiol.* 88 1279–1287. 10.1152/jn.2002.88.3.1279 12205149

[B122] Verdoorn T. A. Burnashev N. Monyer H. Seeburg P. H. Sakmann B. (1991). Structural determinants of ion flow through recombinant glutamate receptor channels. *Science* 252 1715–1718. 10.1126/science.1710829 1710829

[B123] Verret L. Mann E. O. Hang G. B. Barth A. M. I. Cobos I. Ho K. (2012). Inhibitory interneuron deficit links altered network activity and cognitive dysfunction in Alzheimer model. *Cell* 149 708–721. 10.1016/j.cell.2012.02.046 22541439 PMC3375906

[B124] Vieira M. Fernandes J. Burgeiro A. Thomas G. M. Huganir R. L. Duarte C. B. (2010). Excitotoxicity through Ca2+-permeable AMPA receptors requires Ca2+-dependent JNK activation. *Neurobiol. Dis.* 40 645–655. 10.1016/j.nbd.2010.08.008 20708684 PMC3003258

[B125] Vigelsø A. Dybboe R. Hansen C. N. Dela F. Helge J. W. Guadalupe Grau A. (2015). GAPDH and β-actin protein decreases with aging, making Stain-Free technology a superior loading control in Western blotting of human skeletal muscle. *J. Appl. Physiol.* 118 386–394. 10.1152/japplphysiol.00840.2014 25429098

[B126] Vissel B. Royle G. A. Christie B. R. Schiffer H. H. Ghetti A. Tritto T. (2001). The Role of RNA editing of kainate receptors in synaptic plasticity and seizures. *Neuron* 29 217–227. 10.1016/S0896-6273(01)00192-1 11182093

[B127] Wang L. Zhou Y. Chen D. Lee T. H. (2020). Peptidyl-Prolyl Cis/Trans Isomerase Pin1 and Alzheimer’s disease. *Front. Cell Dev. Biol.* 8:355. 10.3389/fcell.2020.00355 32500074 PMC7243138

[B128] Whitesell J. D. Buckley A. R. Knox J. E. Kuan L. Graddis N. Pelos A. (2019). Whole brain imaging reveals distinct spatial patterns of amyloid beta deposition in three mouse models of Alzheimer’s disease. *J. Comp. Neurol.* 527 2122–2145. 10.1002/cne.24555 30311654 PMC8026112

[B129] Wong S. K. Sato S. Lazinski D. W. (2001). Substrate recognition by ADAR1 and ADAR2. *RNA* 7 846–858. 11421361 10.1017/s135583820101007x PMC1370134

[B130] Wright A. Vissel B. (2012). The essential role of AMPA receptor GluR2 subunit RNA editing in the normal and diseased brain. *Front. Mol. Neurosci.* 5:34. 10.3389/fnmol.2012.00034 22514516 PMC3324117

[B131] Wright A. L. Konen L. M. Mockett B. G. Morris G. P. Singh A. Burbano L. E. (2023). The Q/R editing site of AMPA receptor GluA2 subunit acts as an epigenetic switch regulating dendritic spines, neurodegeneration and cognitive deficits in Alzheimer’s disease. *Mol. Neurodegen.* 18:65. 10.1186/s13024-023-00632-5 37759260 PMC10537207

[B132] Wright A. L. Zinn R. Hohensinn B. Konen L. M. Beynon S. B. Tan R. P. (2013). Neuroinflammation and Neuronal Loss Precede Aβ Plaque Deposition in the hAPP-J20 Mouse Model of Alzheimer’s Disease. *PLoS One* 8:e59586. 10.1371/journal.pone.0059586 23560052 PMC3613362

[B133] Xu L.-D. Öhman M. (2019). ADAR1 Editing and its Role in Cancer. *Genes* 10:12. 10.3390/genes10010012 30585209 PMC6356570

[B134] Yamashita T. Kwak S. (2014). The molecular link between inefficient GluA2 Q/R site-RNA editing and TDP-43 pathology in motor neurons of sporadic amyotrophic lateral sclerosis patients. *Brain Res.* 1584 28–38. 10.1016/j.brainres.2013.12.011 24355598

[B135] Yamashita T. Tadami C. Nishimoto Y. Hideyama T. Kimura D. Suzuki T. (2012). RNA editing of the Q/R site of GluA2 in different cultured cell lines that constitutively express different levels of RNA editing enzyme ADAR2. *Neurosci. Res.* 73 42–48. 10.1016/j.neures.2012.02.002 22366356

[B136] Yang B. Hu P. Lin X. Han W. Zhu L. Tan X. (2015). PTBP1 induces ADAR1 p110 isoform expression through IRES-like dependent translation control and influences cell proliferation in gliomas. *Cell. Mol. Life Sci.* 72 4383–4397. 10.1007/s00018-015-1938-7 26047657 PMC11114032

[B137] Yang J. -H. Luo X. Nie Y. Su Y. Zhao Q. Kabir K. (2003). Widespread inosine-containing mRNA in lymphocytes regulated by ADAR1 in response to inflammation. *Immunology* 109 15–23. 10.1046/j.1365-2567.2003.01598.x 12709013 PMC1782949

[B138] Yeung J. H. Y. Walby J. L. Palpagama T. H. Turner C. Waldvogel H. J. Faull R. L. M. (2021). Glutamatergic receptor expression changes in the Alzheimer’s disease hippocampus and entorhinal cortex. *Brain Pathol.* 31 e13005. 10.1111/bpa.13005 34269494 PMC8549033

[B139] Youn D. Royle G. Kolaj M. Vissel B. Randić M. (2008). Enhanced LTP of primary afferent neurotransmission in AMPA receptor GluR2-deficient mice. *PAIN* 136 158–167. 10.1016/j.pain.2007.07.001 17826911

[B140] ZhangX.-X.TianY.WangZ.-T.MaY.-H.TanL.YuJ.-T. (2021). The Epidemiology of Alzheimer’s Disease Modifiable Risk Factors and Prevention. *J. Prev. Alzheimers Dis.* 8 313–321. 10.14283/jpad.2021.15 34101789

[B141] Zhou G.-F. Tang J. Ma Y.-L. Fu X. Liu J.-Y. Yang R.-Z. (2023). ARL6IP1 mediates small-molecule-induced alleviation of Alzheimer pathology through FXR1-dependent BACE1 translation initiation. *Proc. Natl. Acad. Sci. U.S.A.* 120 e2220148120. 10.1073/pnas.2220148120 37216506 PMC10235968

