# Supplementary material for: Quantification of AMPA receptor subunits and RNA editing-related proteins in the J20 mouse model of Alzheimer’s disease by capillary western blotting
Source: Front Mol Neurosci. 2024 Jan 17;16:1338065. doi: 10.3389/fnmol.2023.1338065 (PMC10828003; doi:10.3389/fnmol.2023.1338065)
Supplement: Supplementary file 2 [file Data_Sheet_1.pdf]

# *Supplementary Material*

## 1 Supplementary Figures and Tables

### 1.1 Supplementary Tables

**Supplementary Table 1** Antibodies used in Wes and their recorded kDa peak location

| Target Protein | Primary Antibody | RRID | Peak MW |
|---|---|---|---|
| ADAR1 p110 | Santa Cruz, ADAR1 Antibody [15.8.6] (SC73408) | AB_2222767 | 112-118 kDa |
| ADAR1 p150 | | | 150-160 kDa |
| ADAR2 | Santa Cruz, Anti-ADAR2 (sc-33180) | AB_2222780 | 88-92 kDa |
| ADAR3 | Santa Cruz, ADAR3 Antibody [3.591] (sc-73410) | AB_2222784 | 95-101 kDa |
| GluA1 | Sigma-Aldrich, Anti-Glutamate receptor 1 Antibody (AB1504) | AB_2113602 | 117-121 kDa |
| GluA2 | Cell Signalling, AMPA Receptor 2 (E1L8U) mAb (13607) | AB_2650557 | 115-120 kDa |
| GluA3 | Cell Signalling, AMPA Receptor 3 [D25G9] mAb (5117) | AB_10544796 | 119-122 kDa |
| GluA4 | Cell Signalling, AMPA Receptor 4 [D41A11] XP (8070) | AB_10829469 | 116-125 kDa |
| PIN1 | Cell Signalling, Anti-PIN1 antibody (3722) | AB_10692654 | 22-26 kDa |
| WWP2 | Invitrogen, WWP2 Polyclonal Antibody (A302-936A) | AB_10663490 | 110-120 kDa |
| FXR1 | Abcam, Anti-FXR1 antibody [EPR7932] (ab129089) | AB_11154960 | 70-74 kDa |
| CREB1 | ThermoFisher, CREB Monoclonal Antibody [LB 9] (350900) | AB_2533194 | 55-60 kDa |

## 1.2 Supplementary Figures

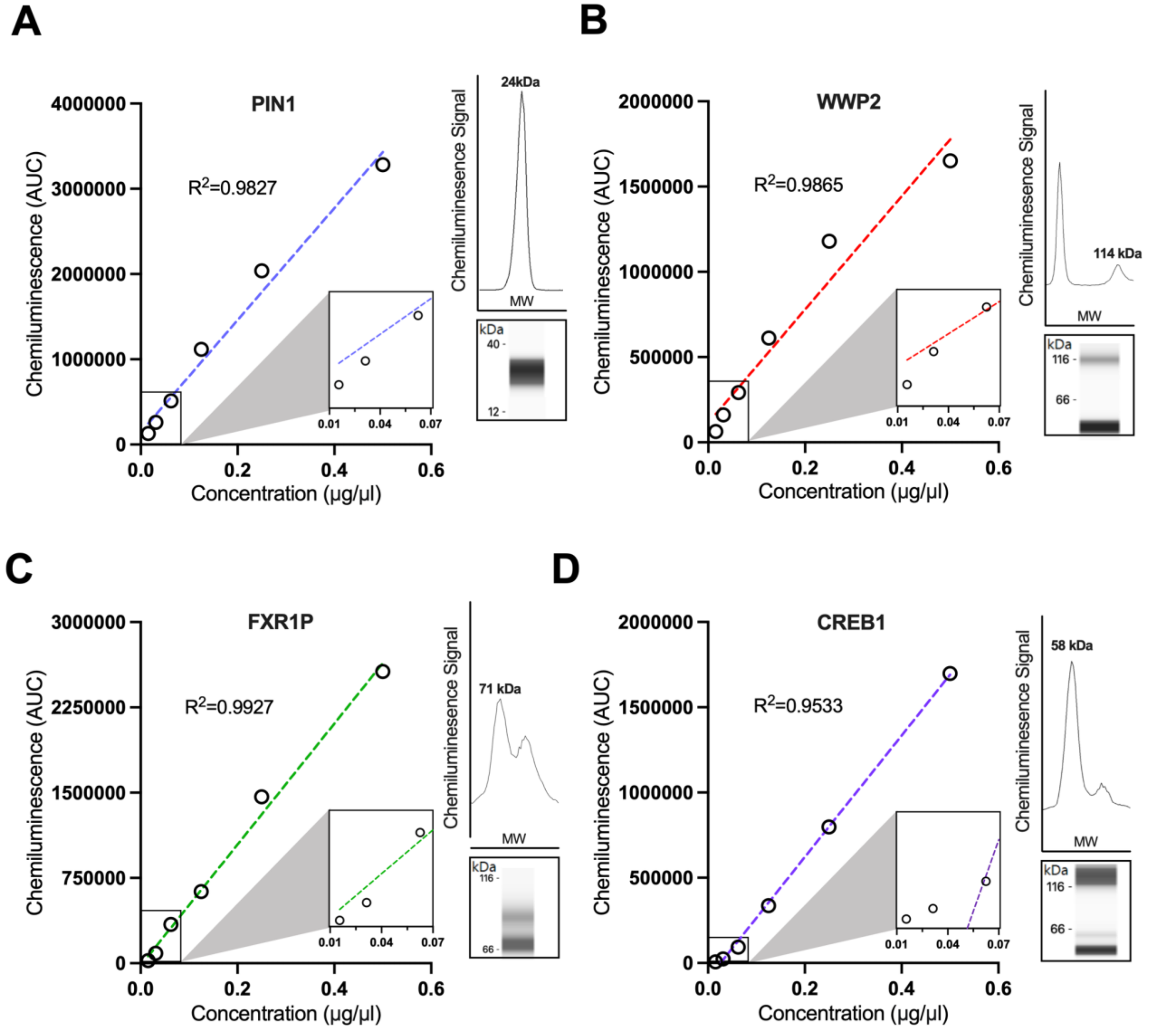

**Supplementary Figure 1.** Optimisation of proteins linked to ADAR editing activity. Simple linear regression analysis of chemiluminescence signals obtained from a two-fold protein dilution series for PIN1 (**A;** $r^2$=0.9882), WWP2 (**B;** $r^2$=0.9724), FXR1P (**C;** $r^2$=0.9983) and CREB1 (**D;** $r^2$=0.9983). Insets situated right of all graphs display the electropherogram peak (above) with a computer-generated blot (below) for the relevant target protein analysed. n=1 for all dilutions.

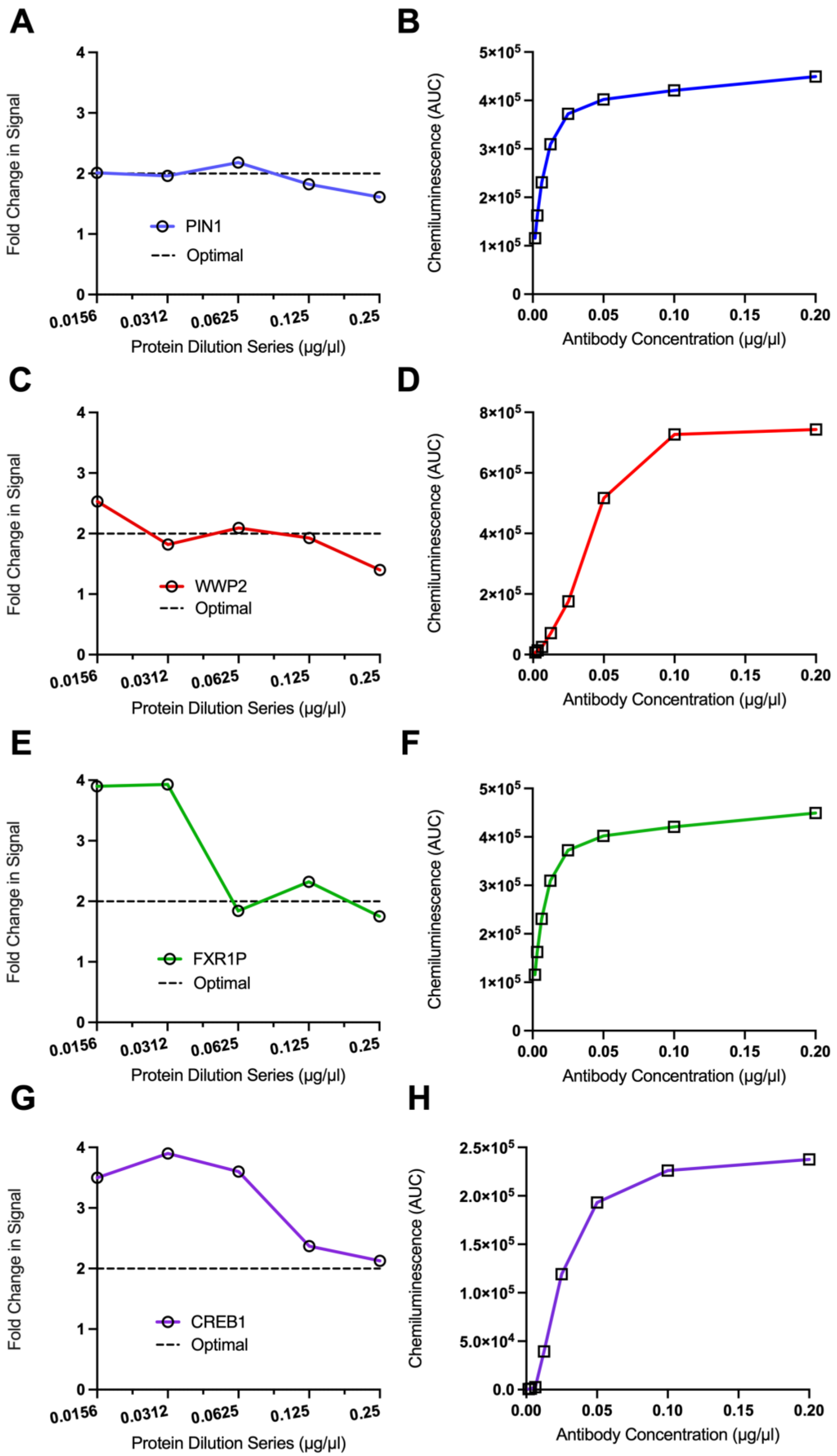
A
B
C
D
E
F
G
H
Fold Change in Signal
Protein Dilution Series (µg/µl)
0.0156
0.0312
0.0625
0.125
0.25
PIN1
WWP2
FXR1P
CREB1
Optimal
Chemiluminescence (AUC)
Antibody Concentration (µg/µl)
0.00
0.05
0.10
0.15
0.20

**Supplementary Figure 2.** Optimisation of proteins and antibodies linked to ADAR editing activity. Analysis of fold changes in chemiluminescence signals across a two-fold dilution series for PIN1 **(A)**, WWP2 **(C)**, FXR1P **(E)** and CREB1 **(G)** (n=1, Dotted line represents theoretical optimal result for visual comparison). Determination of relative plateau in chemiluminescence signal of target antibody over a two-fold antibody dilution series for PIN1 **(B)**, WWP2 **(D)**, FXR1P **(F)** and CREB1 **(H)**. n=1 for all dilutions.

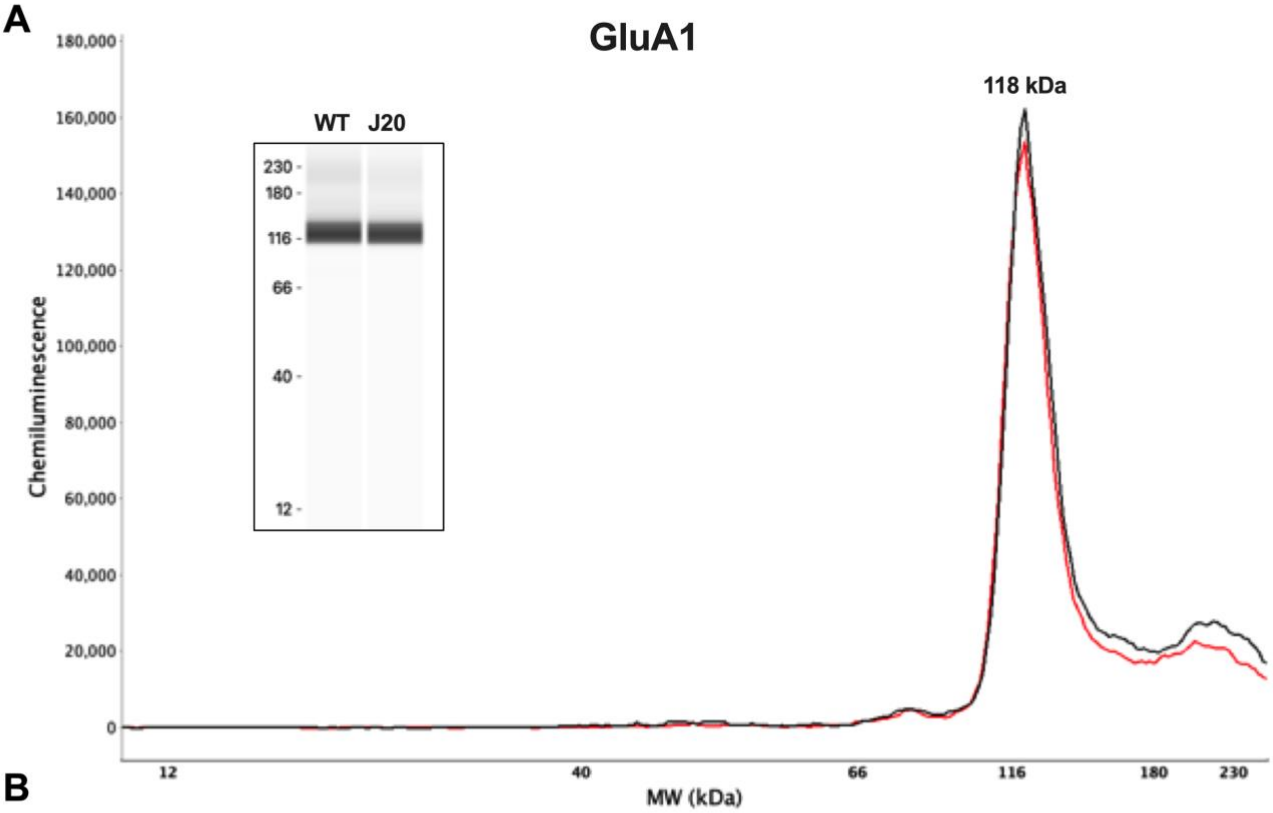
A
GluA1
118 kDa
WT J20
230
180
116
66
40
12
Chemiluminescence
180,000
160,000
140,000
120,000
100,000
80,000
60,000
40,000
20,000
0
12
40
66
116
180
230
MW (kDa)

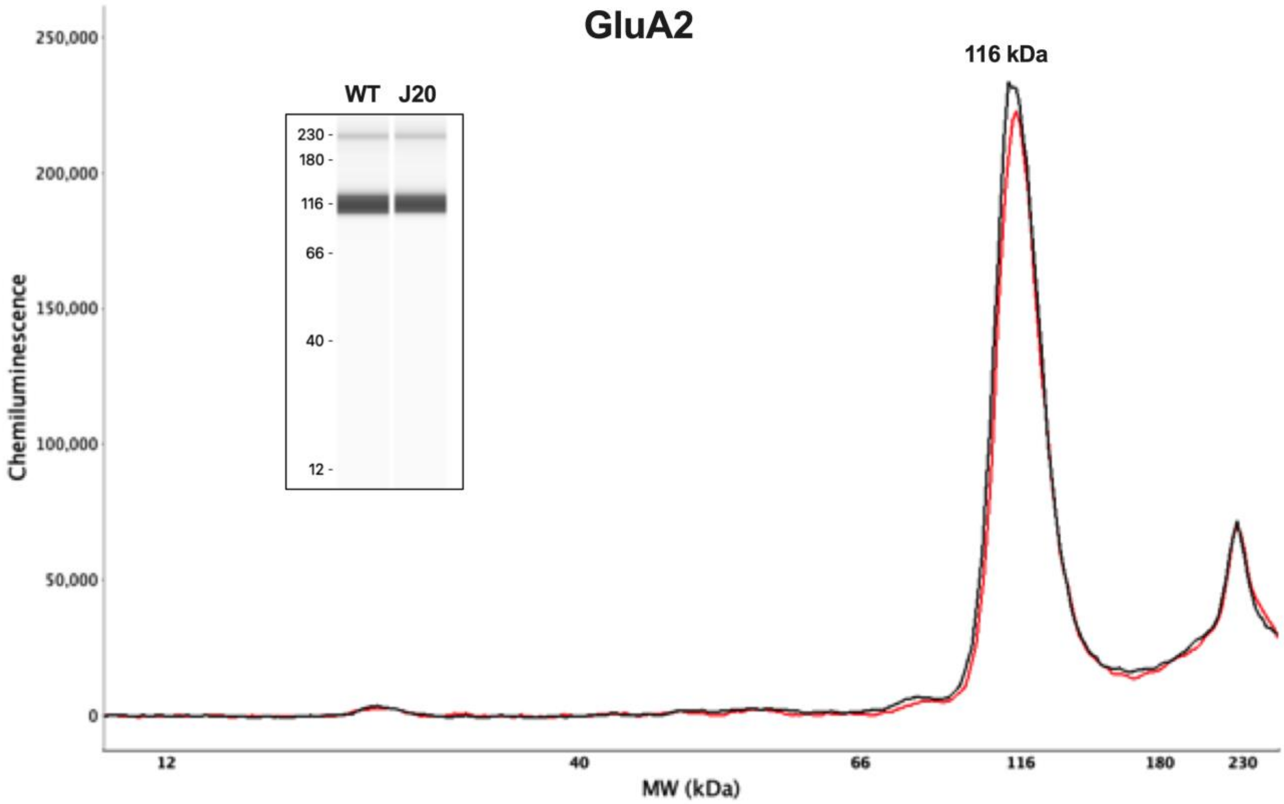
B
GluA2
116 kDa
WT J20
230
180
116
66
40
12
Chemiluminescence
250,000
200,000
150,000
100,000
50,000
0
12
40
66
116
180
230
MW (kDa)

**Supplementary Figure 3.** Example of uncropped chemiluminescent electropherogram traces of GluA1 **(A)** and GluA2 **(B)** in aged J20 (red lines) and WT (black lines) hippocampal tissue. Insets situated left of all graphs display a computer-generated uncropped blot for the related target proteins analysed.

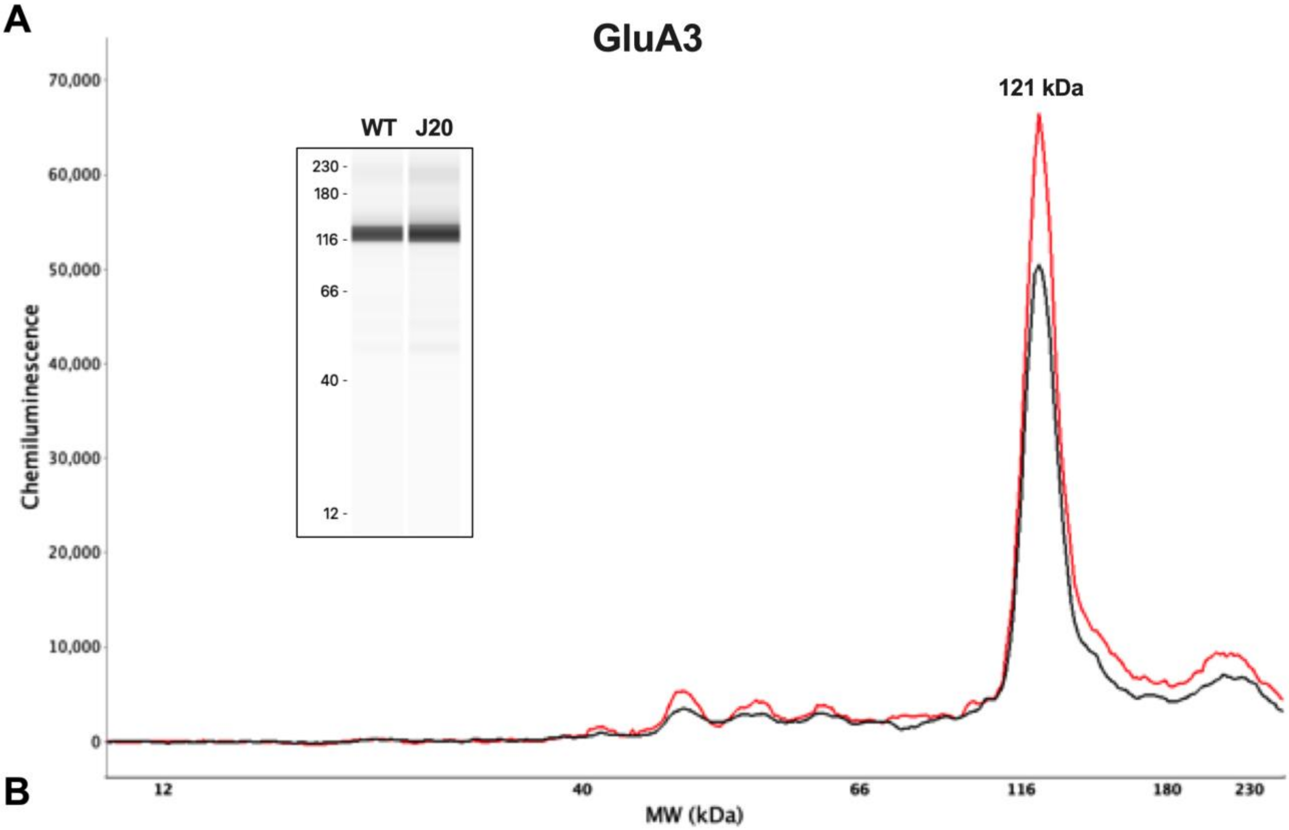
A
GluA3
121 kDa
WT J20
230 -
180 -
116 -
66 -
40 -
12 -
70,000
60,000
50,000
40,000
30,000
20,000
10,000
0
Chemiluminescence
12
40
66
116
180
230
MW (kDa)

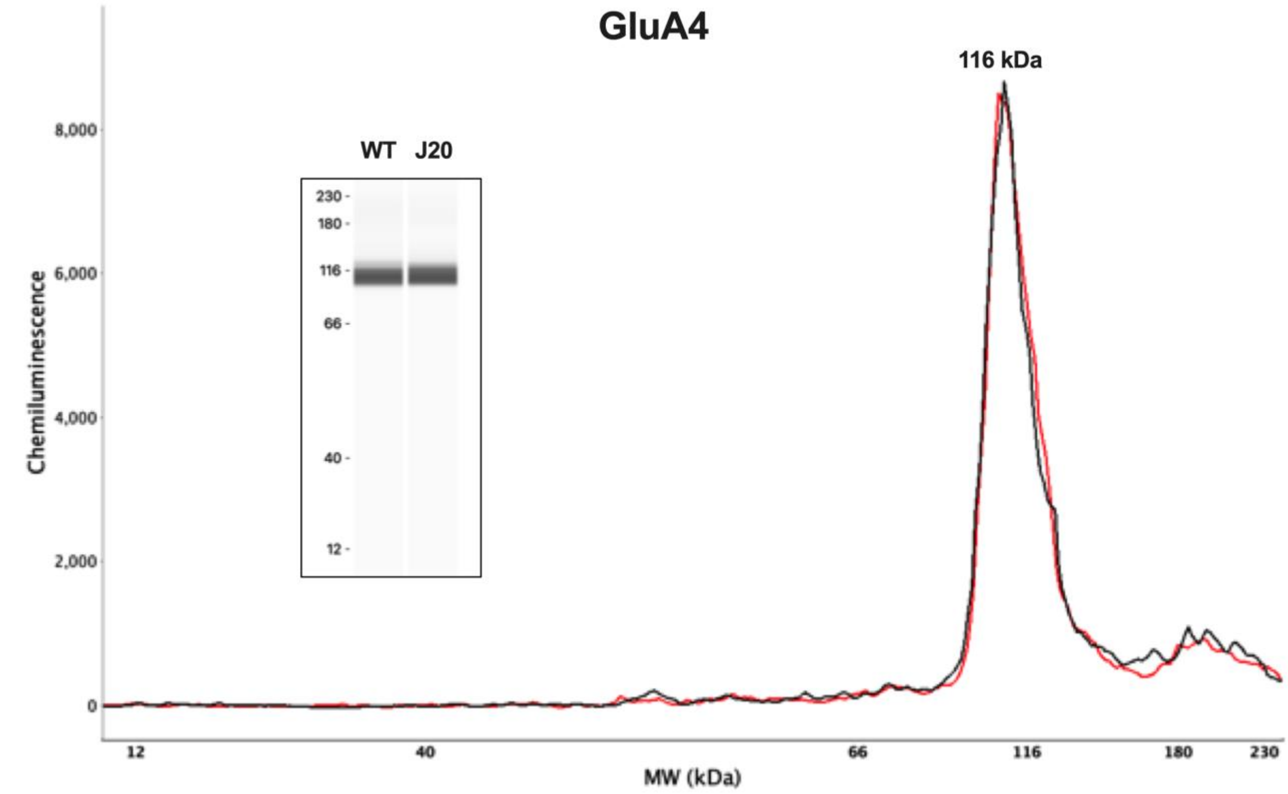
B
GluA4
116 kDa
WT J20
230 -
180 -
116 -
66 -
40 -
12 -
8,000
6,000
4,000
2,000
0
Chemiluminescence
12
40
66
116
180
230
MW (kDa)

**Supplementary Figure 4.** Example of uncropped chemiluminescent electropherogram traces of GluA3 **(A)** and GluA4 **(B)** in aged J20 (red lines) and WT (black lines) hippocampal tissue. Insets situated left of all graphs display a computer-generated uncropped blot for the related target proteins analysed.

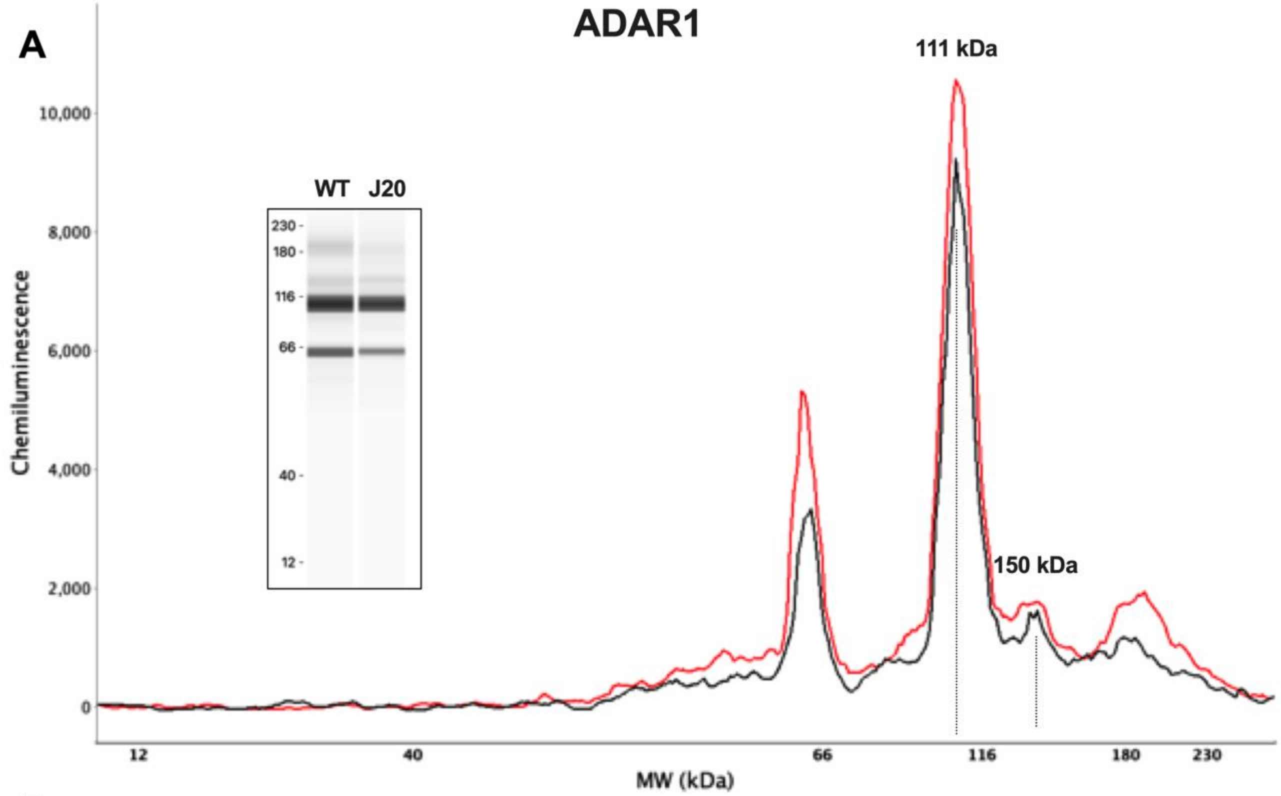
A
ADAR1
111 kDa
150 kDa
WT J20
230
180
116
66
40
12
Chemiluminescence
10,000
8,000
6,000
4,000
2,000
0
12
40
66
116
180
230
MW (kDa)

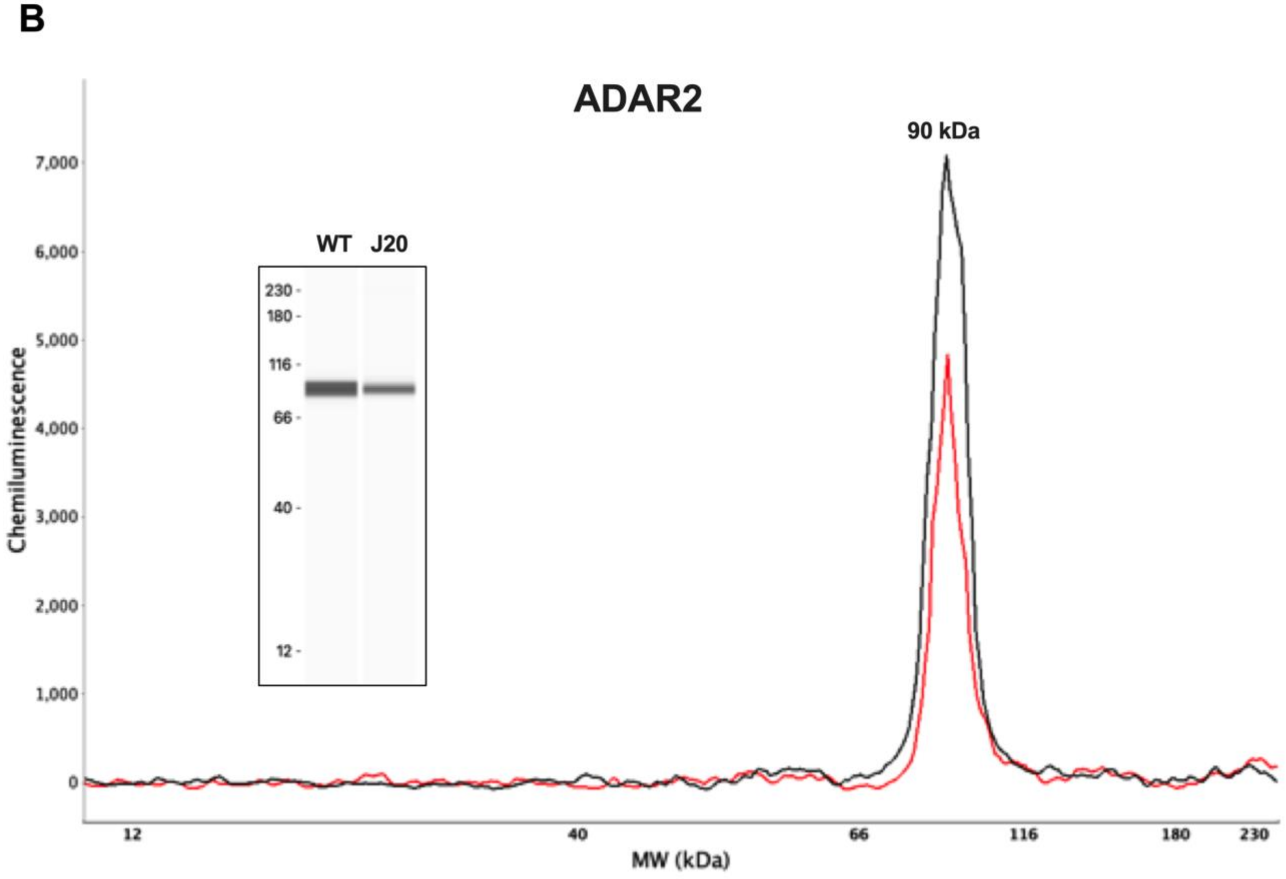
B
ADAR2
90 kDa
WT J20
230
180
116
66
40
12
Chemiluminescence
7,000
6,000
5,000
4,000
3,000
2,000
1,000
0
12
40
66
116
180
230
MW (kDa)

**Supplementary Figure 5.** Example of uncropped chemiluminescent electropherogram traces of ADAR1 p110/p150 isoforms **(A)** and ADAR2 **(B)** in aged J20 (red lines) and WT (black lines) hippocampal tissue. Insets situated left of all graphs display a computer-generated uncropped blot for the related target proteins analysed. Peaks of interest are identified with dotted line.

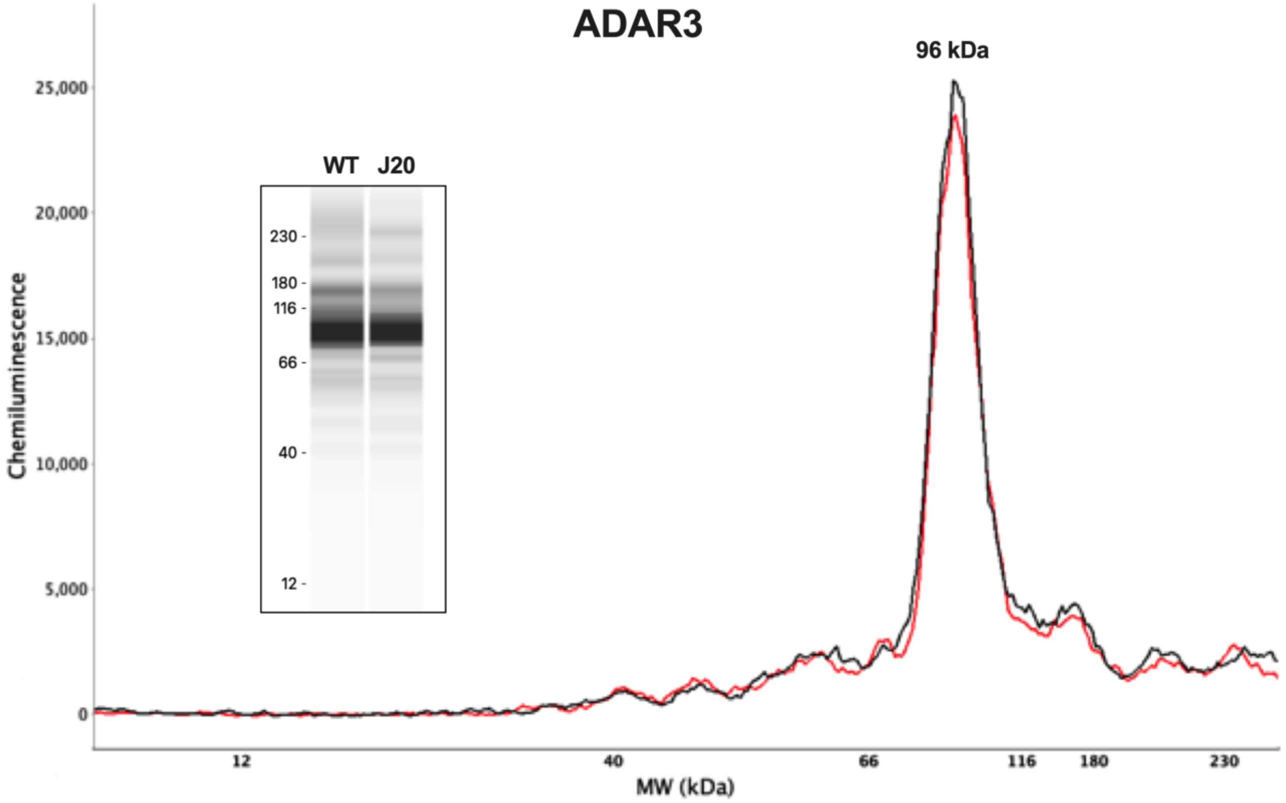
ADAR3
96 kDa
WT J20
230 -
180 -
116 -
66 -
40 -
12 -
Chemiluminescence
25,000
20,000
15,000
10,000
5,000
0
12
40
66
116
180
230
MW (kDa)

**Supplementary Figure 6.** Example of uncropped chemiluminescent electropherogram trace of ADAR3 in aged J20 (red lines) and WT (black lines) hippocampal tissue. Insets situated left of all graphs display a computer-generated uncropped blot for the related target proteins analysed.

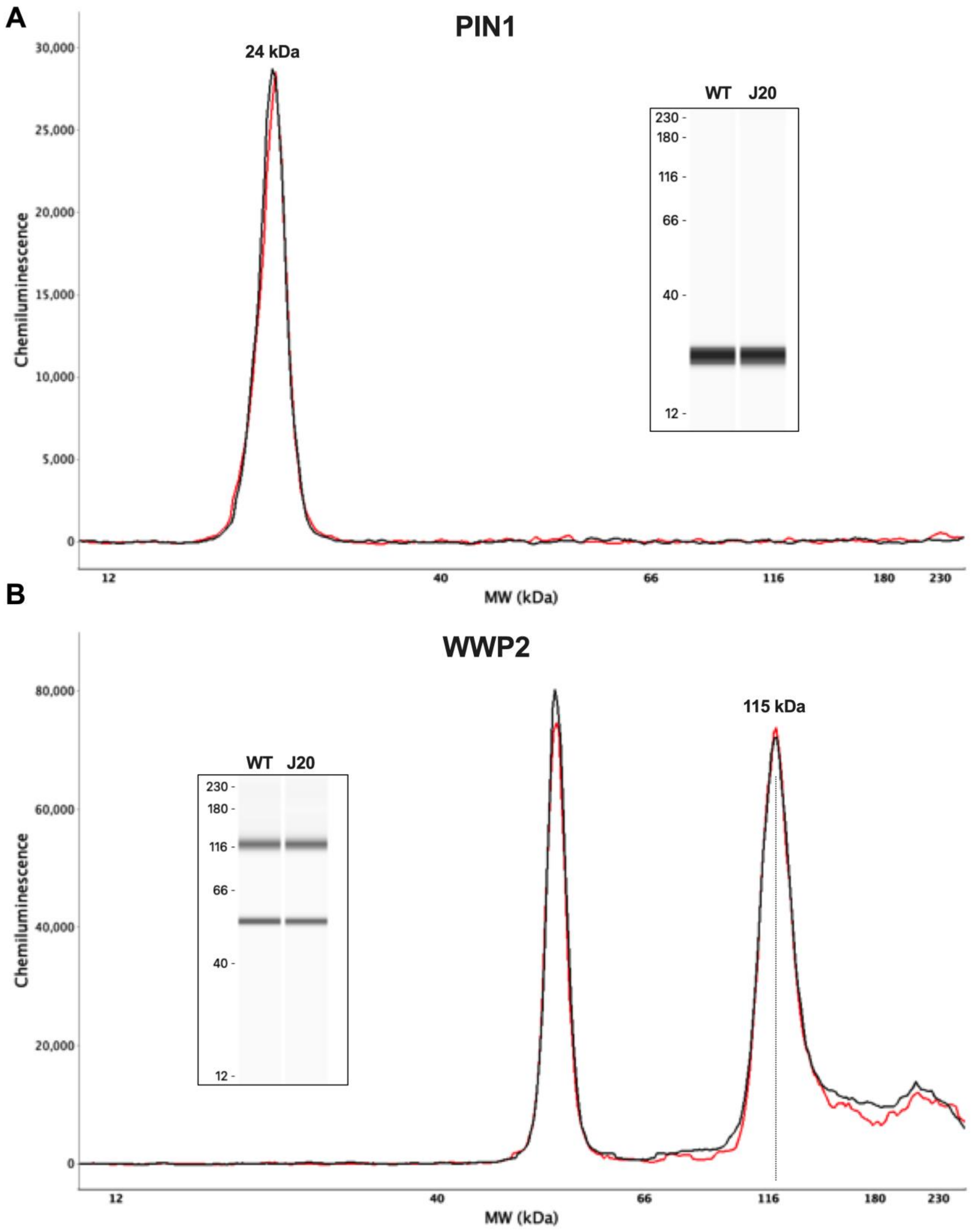
A
PIN1
24 kDa
Chemiluminescence
30,000
25,000
20,000
15,000
10,000
5,000
0
WT J20
230 -
180 -
116 -
66 -
40 -
12 -
12
40
66
116
180
230
MW (kDa)
B
WWP2
115 kDa
80,000
60,000
40,000
20,000
0
Chemiluminescence
WT J20
230 -
180 -
116 -
66 -
40 -
12 -
12
40
66
116
180
230
MW (kDa)

**Supplementary Figure 7.** Example of uncropped chemiluminescent electropherogram traces of PIN1 **(A)** and WWP2 **(B)** in aged J20 (red lines) and WT (black lines) hippocampal tissue. Insets situated right and left of graphs respectively display a computer-generated uncropped blot for the related target proteins analysed. Peaks of interest are identified with dotted line.

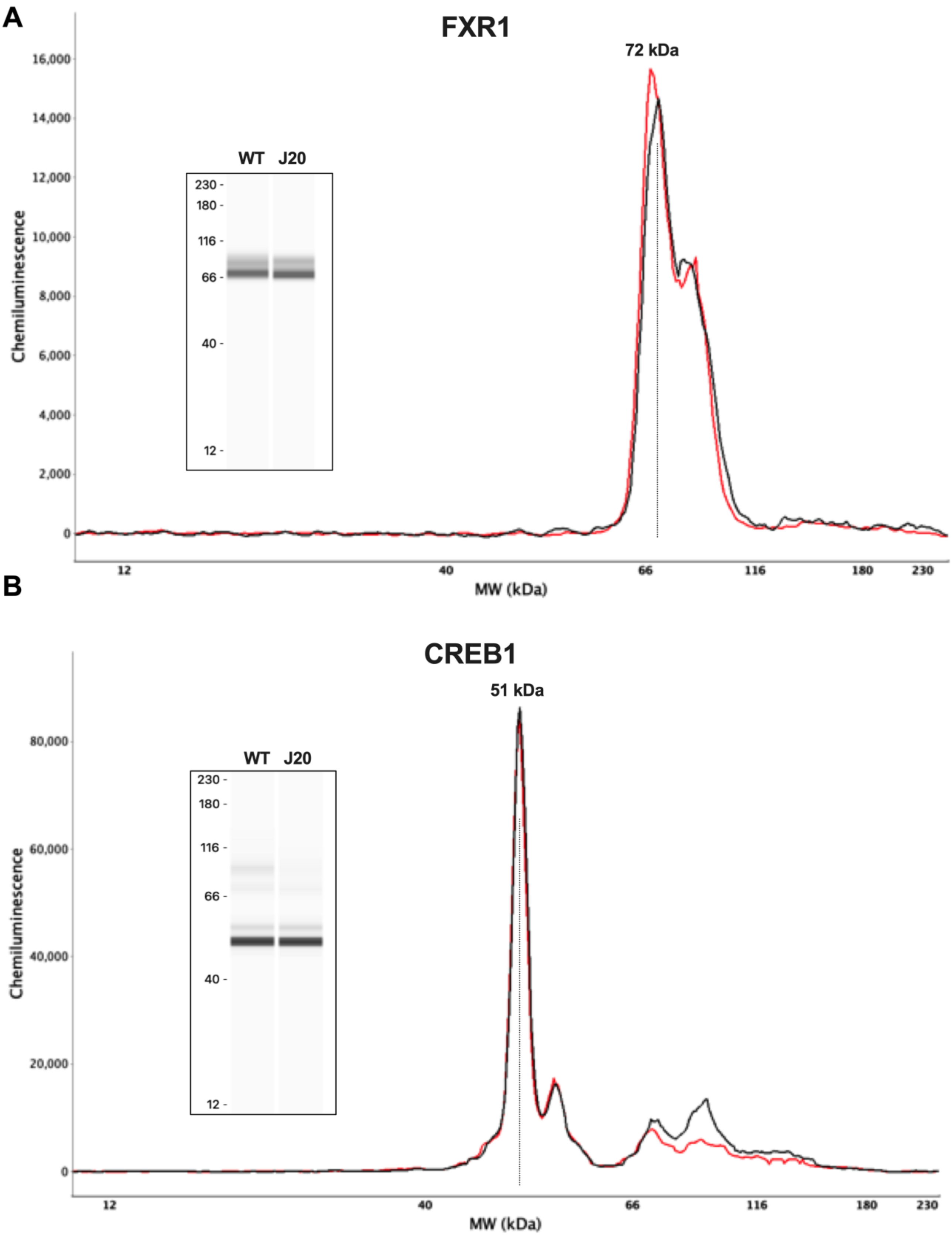
A
FXR1
72 kDa
Chemiluminescence
16,000
14,000
12,000
10,000
8,000
6,000
4,000
2,000
0
WT J20
230 -
180 -
116 -
66 -
40 -
12 -
12
40
66
116
180
230
MW (kDa)
B
CREB1
51 kDa
Chemiluminescence
80,000
60,000
40,000
20,000
0
WT J20
230 -
180 -
116 -
66 -
40 -
12 -
12
40
66
116
180
230
MW (kDa)

**Supplementary Figure 8.** Example of uncropped chemiluminescent electropherogram traces of FXR1P **(A)** and CREB1**(B)** in aged J20 (red lines) and WT (black lines) hippocampal tissue. Insets situated left of all graphs display a computer-generated uncropped blot for the related target proteins analysed. Peaks of interest are identified with dotted line.

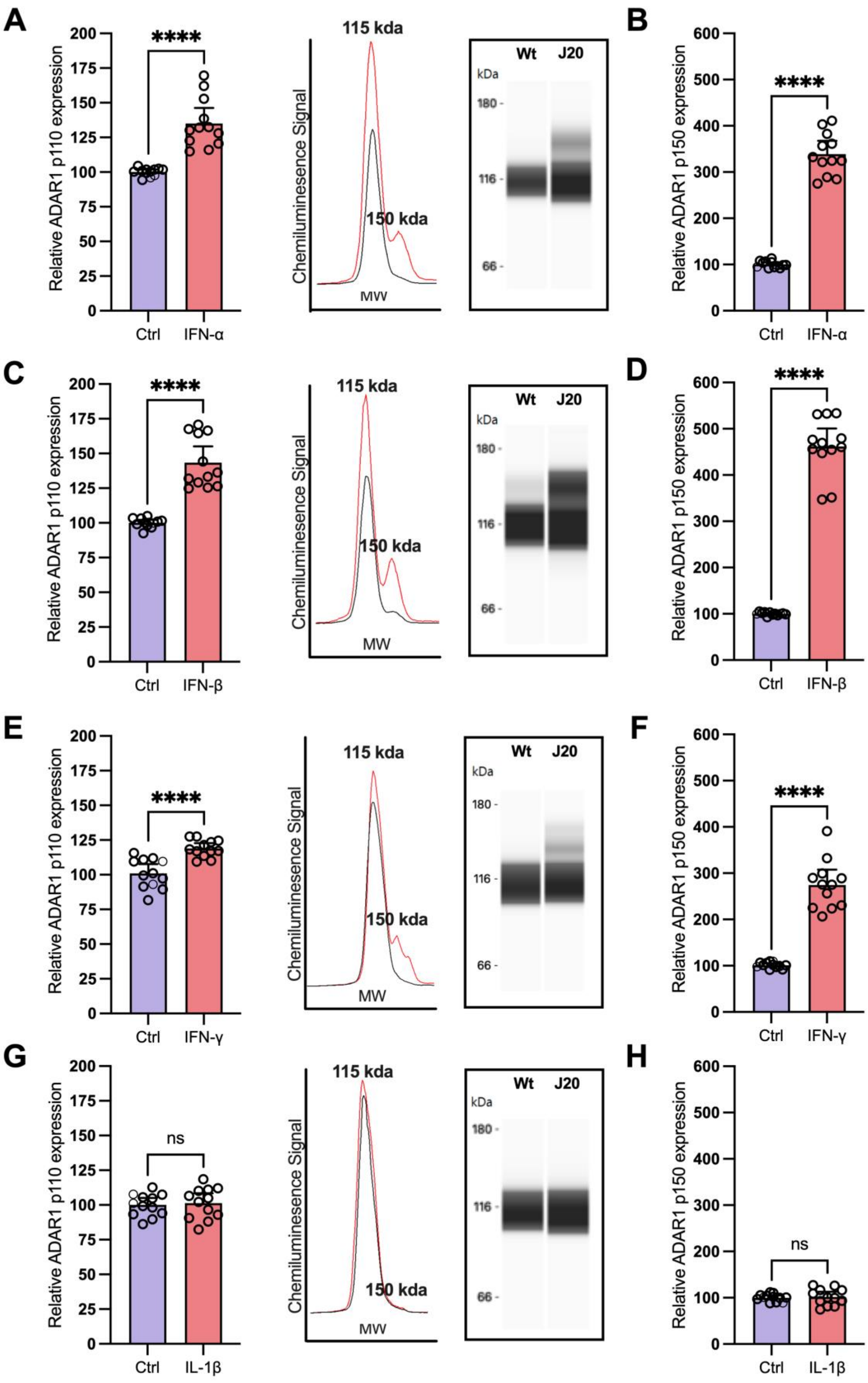
A
Relative ADAR1 p110 expression
****
Ctrl
IFN-α
115 kda
150 kda
Chemiluminesence Signal
MW
Wt
J20
kDa
180
116
66
B
Relative ADAR1 p150 expression
****
Ctrl
IFN-α
C
Relative ADAR1 p110 expression
****
Ctrl
IFN-β
115 kda
150 kda
Chemiluminesence Signal
MW
Wt
J20
kDa
180
116
66
D
Relative ADAR1 p150 expression
****
Ctrl
IFN-β
E
Relative ADAR1 p110 expression
****
Ctrl
IFN-γ
115 kda
150 kda
Chemiluminesence Signal
MW
Wt
J20
kDa
180
116
66
F
Relative ADAR1 p150 expression
****
Ctrl
IFN-γ
G
Relative ADAR1 p110 expression
ns
Ctrl
IL-1β
115 kda
150 kda
Chemiluminesence Signal
MW
Wt
J20
kDa
180
116
66
H
Relative ADAR1 p150 expression
ns
Ctrl
IL-1β

**Supplementary Figure 9.** Analysis of ADAR1 isoform expression following interferon and IL-1B treatment on SH-SY5Y cell line. Relative expression of ADAR1 p110 and ADAR1 p150 following IFN-α (**A-B;** unpaired t-test), IFN-β (**C-D;** unpaired t-test), IFN-γ (**E-F;** unpaired t-test) treatment was upregulated in SH-SY5Y cells but remained unchanged following IL-1β (**G-H;** unpaired t-test) treatment (n=12 for all groups). Insets situated right and left of all graphs display a computer-generated blot for the related target protein analysed. ****$p<0.0001$. ns=not statistically significant. All figures are mean ± SD.

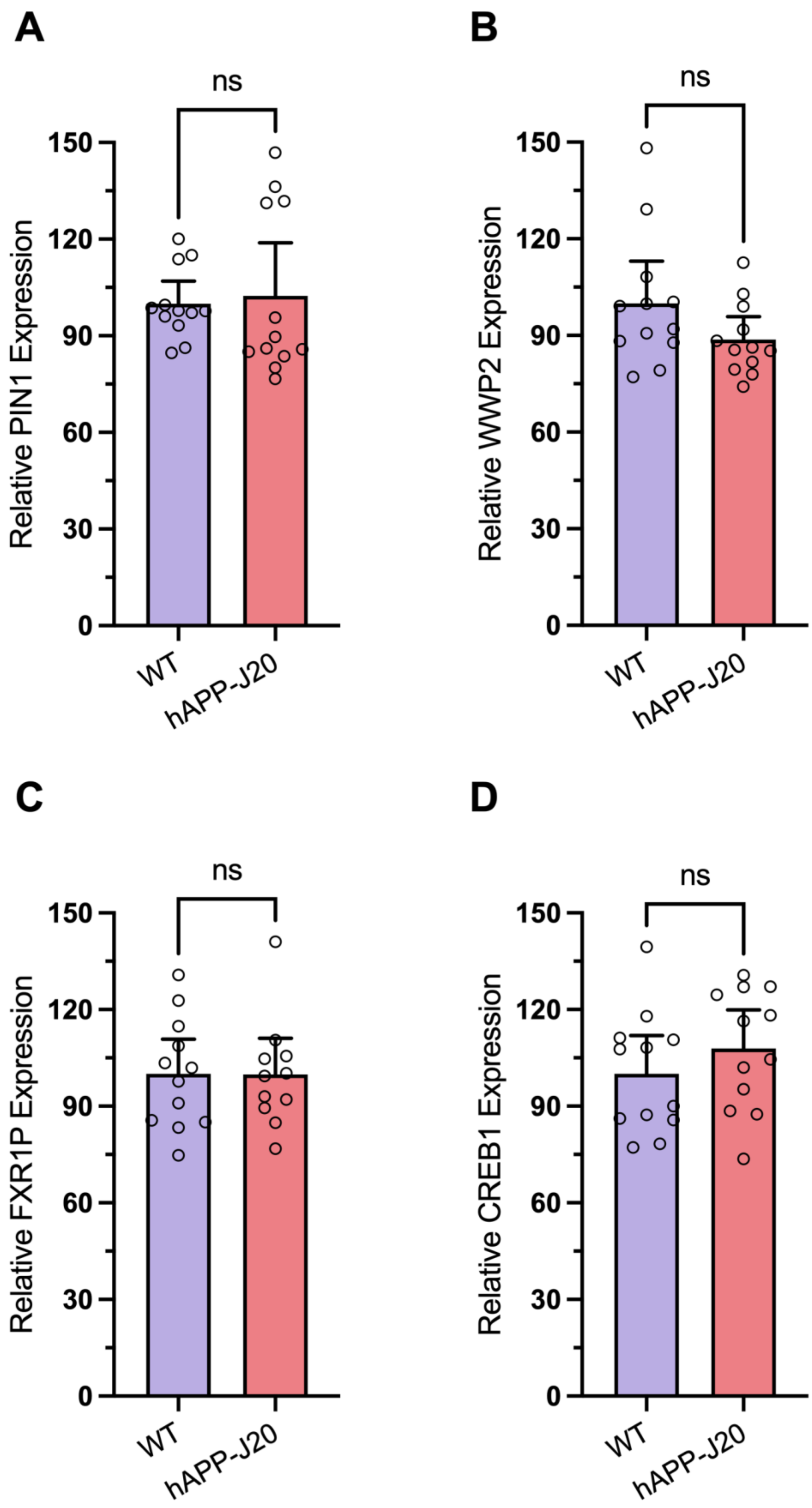
A
ns
Relative PIN1 Expression
0
30
60
90
120
150
WT
hAPP-J20
B
ns
Relative WWP2 Expression
0
30
60
90
120
150
WT
hAPP-J20
C
ns
Relative FXR1P Expression
0
30
60
90
120
150
WT
hAPP-J20
D
ns
Relative CREB1 Expression
0
30
60
90
120
150
WT
hAPP-J20

**Supplementary Figure 10.** Analysis of proteins linked to ADAR editing activity in aged J20 and WT hippocampal tissue. Relative expression of PIN1 (**A;** unpaired t-test *p*=0.7700), WWP2 (**B;** Mann Whitney *p*=0.1277), FXR1P (**C;** unpaired t-test *p*=0.9794) and CREB1 (**D;** unpaired t-test *p*=0.3111) are unchanged in the hippocampus of J20 mice when compared to age-matched WT littermates (n=12 for all groups). ns=not statistically significant. All figures are mean ± SD.

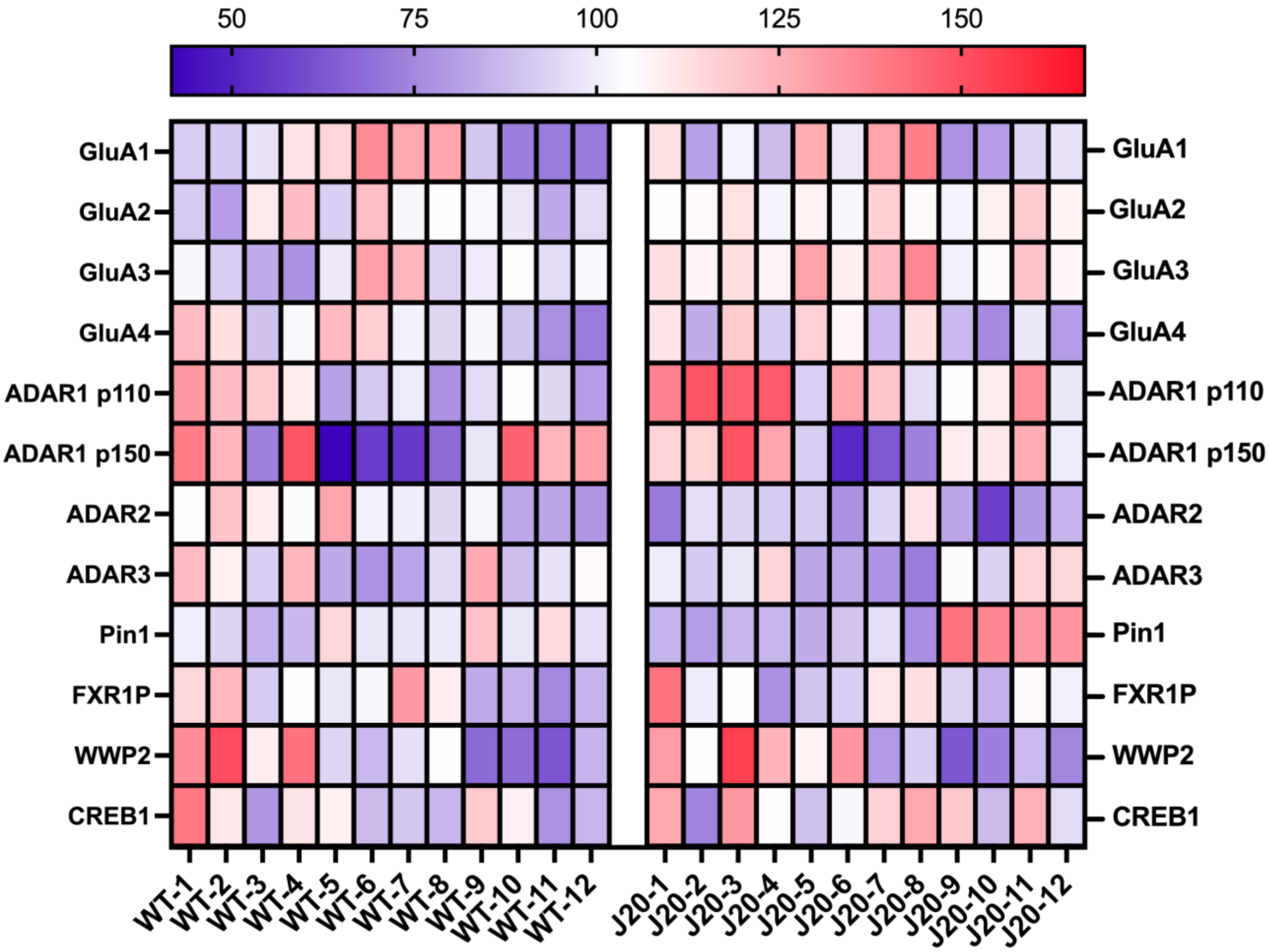
50
75
100
125
150
GluA1
GluA2
GluA3
GluA4
ADAR1 p110
ADAR1 p150
ADAR2
ADAR3
Pin1
FXR1P
WWP2
CREB1
WT-1
WT-2
WT-3
WT-4
WT-5
WT-6
WT-7
WT-8
WT-9
WT-10
WT-11
WT-12
J20-1
J20-2
J20-3
J20-4
J20-5
J20-6
J20-7
J20-8
J20-9
J20-10
J20-11
J20-12

**Supplementary Figure 11.** Heatmap of the relative expression of all protein targets across individual mouse hippocampi in WT and aged J20 cohorts. Upregulation target protein above the normalised 100% are indicated with increasing red colouration and downregulations are indicated with increasing blue colouration.
